# Micro-evolution of three *Streptococcus* species: selection, antigenic variation, and horizontal gene inflow

**DOI:** 10.1186/s12862-019-1403-6

**Published:** 2019-03-27

**Authors:** Pavel V. Shelyakin, Olga O. Bochkareva, Anna A. Karan, Mikhail S. Gelfand

**Affiliations:** 1 Vavilov Institute of General Genetics Russian Academy of Sciences, Gubkina str. 3, Moscow, 119991 Russia; 2 Kharkevich Institute for Information Transmission Problems, 19, Bolshoy Karetny per., Moscow, 127051 Russia; 3 Center of Life Sciences, Skolkovo Institute of Science and Technology, Moscow, Russia; 4 Faculty of Bioengineering and Bioinformatics, Lomonosov Moscow State University, Moscow, Russia; 5 Faculty of Computer Science, Higher School of Economics, Moscow, Russia

**Keywords:** *Streptococcus*, Pan-genome, Genomic rearrangements, Antigen variation, Gene inflow, Selection in upstream regions

## Abstract

**Background:**

The genus *Streptococcus* comprises pathogens that strongly influence the health of humans and animals. Genome sequencing of multiple *Streptococcus* strains demonstrated high variability in gene content and order even in closely related strains of the same species and created a newly emerged object for genomic analysis, the pan-genome. Here we analysed the genome evolution of 25 strains of *Streptococcus suis*, 50 strains of *Streptococcus pyogenes* and 28 strains of *Streptococcus pneumoniae*.

**Results:**

Fractions of the pan-genome, unique, periphery, and universal genes differ in size, functional composition, the level of nucleotide substitutions, and predisposition to horizontal gene transfer and genomic rearrangements. The density of substitutions in intergenic regions appears to be correlated with selection acting on adjacent genes, implying that more conserved genes tend to have more conserved regulatory regions.

The total pan-genome of the genus is open, but only due to strain-specific genes, whereas other pan-genome fractions reach saturation. We have identified the set of genes with phylogenies inconsistent with species and non-conserved location in the chromosome; these genes are rare in at least one species and have likely experienced recent horizontal transfer between species. The strain-specific fraction is enriched with mobile elements and hypothetical proteins, but also contains a number of candidate virulence-related genes, so it may have a strong impact on adaptability and pathogenicity.

Mapping the rearrangements to the phylogenetic tree revealed large parallel inversions in all species. A parallel inversion of length 15 kB with breakpoints formed by genes encoding surface antigen proteins PhtD and PhtB in *S. pneumoniae* leads to replacement of gene fragments that likely indicates the action of an antigen variation mechanism.

**Conclusions:**

Members of genus *Streptococcus* have a highly dynamic, open pan-genome, that potentially confers them with the ability to adapt to changing environmental conditions, i.e. antibiotic resistance or transmission between different hosts. Hence, integrated analysis of all aspects of genome evolution is important for the identification of potential pathogens and design of drugs and vaccines.

**Electronic supplementary material:**

The online version of this article (10.1186/s12862-019-1403-6) contains supplementary material, which is available to authorized users.

## Background

The genus *Streptococcus* are Gram-positive bacteria that exert strong influence on the health of humans and animals. In particular, *Streptococcus pneumoniae*, normally a commensal from the nasopharynx microflora, at the same time is responsible for most pneumonia cases and is second only to *Mycobacterium tuberculosis* as a cause of mortality from bacterial infection worldwide [1]. *Streptococcus pyogenes* is among the top ten of bacterial causes of human mortality worldwide [2, 3], and due to the molecular mimicry with heart and brain cells causes severe autoimmune sequelae like rheumatic fever [4] and, possibly, autoimmune neuropsychiatric disorders [5]. *Streptococcus suis* rarely causes disease in human, but is one of the most important swine pathogens [6].

Sequencing of multiple strains of one species has demonstrated that the genome of any single strain does not reflect the genetic variability of the species, as two strains may differ by 20–35% of the gene content [7]. The concept of pan-genome was introduced to represent the total set of genes observed in genomes of strains assigned to a given species [7–9]. The pan-genome consists of core genes, present in all sequenced strains, dispensable, or periphery, genes, present in a subset of strains, and unique, strain-specific genes. The pan-genome is said to be open if upon addition of new strains its size continues to grow, or closed, if at some point it saturates [7].

Fractions of the pan-genome may differ not only in size, but also in the functional composition [10]. In general, core genes encode housekeeping functions, while dispensable and unique genes confer selective advantages such as adaptation to particular niches, e.g. colonization of different hosts for pathogens, or antibiotic resistance [11]. So one may expect that genes from different fractions of the pan-genome evolve in different modes, including gene gain/loss rate, frequency of horizontal gene transfer, and selective pressure [12, 13].

A consequence of the highly dynamic nature of bacterial genomes is frequent genomic rearrangements. Large inversions across the replication axis, deletions and insertions have been observed in *S. pneumoniae* [14, 15], *S. suis* [16, 17] and *S. pyogenes* [18]. The inversions have been suggested to rebalance the chromosomal architecture affected by insertions of large DNA segments [14]. The majority of these rearrangements occur at genome areas encoding transposases. Other genomic rearrangements occur at rRNA operons or sites encoding phage integrases and/or phage-related proteins.

Genome arrangement may have profound effects on a bacterial phenotype. Rearrangements can disrupt genes, create new genes by fusion of gene parts, or change gene expression. One example of such inversions is truncation of the so-called *srtF* pilus island in *S. pneumoniae* NSUI060 [19]. In *S. pyogenes* M23ND, genomic rearrangements resulted in re-clustering of a broad set of *CovRS*-regulated, actively transcribed genes, including virulence factors and metabolic genes, to the same leading strand. This may provide a potential advantage by creating spatial proximity to the transcription complexes, which may contain the global transcriptional regulator, *CovRS*, and RNA polymerases, in turn allowing for efficient transcription of the genes required for growth, virulence, and persistence [20].

Here we describe a comprehensive pan-genomic analysis of *S. pneumoniae*, *S. pyogenes*, and *S. suis* strains with integrated analysis of their genome evolution. The paper is organized as follows. First, we describe and functionally characterize the pan-genome and then use the results of this analysis to detect variations in selection regimes for genes and intergenic regions from different pan-genome fractions. Next, we focus on genome rearrangements revealing large parallel inversions in all studied species and make a prediction of the antigenic variation of histidine triad protein PhtD in *S. pneumoniae*. Finally, we use the gene order data to identify and functionally characterize the fraction of genes horizontally transferred after the divergence of the studied species and further spreading between the strains.

## Methods

### Genome sequences

The selection of the species was based on the number of available strain genomes. We analyzed 25 strains of *Streptococcus suis*, 50 strains of *Streptococcus pyogenes*, and 28 strains of *Streptococcus pneumoniae*, all available complete genomes as of June 2016 (Additional files 1: Table S1 and 2: Figure S1). The complete genomes were downloaded from the GenBank [21]. For all but two genomes (*Streptococcus pyogenes* STAB901 and *Streptococcus pyogenes* MTB313) the GenBank annotation coincides with that of the NCBI Refseq database.

### Construction of orthologous groups (OGs)

We constructed orthologous groups using Proteinortho v5.13 with the default parameters [22]. Each gene was thus assigned to an orthologous group or labeled as a singleton. The size of a pan-genome was estimated with the Chao algorithm from the Micropan R-package [23].

### Assignment of Gene Ontology (GO) terms to orthologous groups

To assign GO terms to genes, we used Interproscan [24]. A GO term was assigned to an orthologous group, if it was assigned to at least 90% of genes in this group. To determine overrepresented functional categories, we used GOstat [25]. The fit by theoretical models was estimated using the Akaike information criterion (AIC) [26].

### Assignment of KEGG Orthology (KO) categories to orthologous groups

Initially, we assigned KO categories to genes with GhostKOALA [27]. Then a KO category was assigned to an orthologous group, if it was assigned to at least 90% of genes in this group. KO terms were divided into supercategories “Genetic Information Processing”, “Metabolism”, “Cellular Processes”, “Environmental Information Processing”, and “other” based on the KEGG hierarchy classification.

### Prediction of virulence-related orthologous groups

We found virulence-related genes with MP3 (threshold 0.2) [28] that combined a support vector machine classifier trained on virulence factors from MvirDB [29] and a hidden Markov model classifier based on Pfam domains present in virulence factors. Orthologous group was considered virulence-related if at least 10% of its members were predicted to be virulence-related. To predict potential prophages, we used web server PHAST [30].

### *pN*/*pS* calculation

To estimate the number of synonymous (*pS*) and nonsynonymous (*pN*) polymorphisms, we aligned amino acid sequences of proteins using MUSCLE [31] and then reconstituted the corresponding nucleotide alignment. Then we calculated *pN* and *pS* using the KaKs-Calculator Toolbox v2.0 with the Modified version of the Yang-Nielsen (MYN) method [32]. Multiple substitutions were accounted for using the Jukes-Cantor correction [33]. For these calculations, we considered different *Streptococcus* species separately. While homologous recombination clearly is important for the *Streptococcus* evolution, in case of pairs of very close genomes, homologous recombination would affect synonymous and non-synonymous substitution at the same degree. For each species and each orthologous group not containing paralogos, we performed pairwise comparisons of all strains and assigned the median *pN*/*pS* ratio to this group.

### Selection in intergenic regions

We extracted intergenic regions from.gbk files downloaded from the NCBI Genome database. We removed intergenic regions shorter then 50 bp. Out of the remaining intergenic regions we constructed the sample of upstream fragments in the following way. We extracted 100 bp upstream fragments for all intergenic regions longer than 100 bp [34, 35]. For intergenic regions shorter than 100 bp its complete sequence was considered as an upstream fragment.

We estimated the fraction of positions under negative selection in two ways. To assess the correlation between the level of conservation in intergenic region and universality of the respective genes, we simply calculated substitutions in upstream fragments. Specifically, we considered all pairs of strains from one species, extracted aligned upstream fragments of orthologous genes from the multiple genome alignment, and counted nucleotide substitutions with the Jukes-Cantor correction. The same approach was used to compare the conservation level in univeral regions and regions deleted in some strains.

To estimate the overall selection pressure in intergenic regions, we applied the method from [36] to calculate the fraction of positions under negative selection by comparing conservation statistics of multiple sequence alignments of orthologous upstream fragments from strains of two closely related species.

### Detection and analysis of large insertions/deletions (indels)

In orthologous upstream fragments, we considered all indels of length at least six nucleotides, observed in at least two strains, and not located at the alignments termini (to reduce the bias from misalignment of fragment termini and varying length of upstream regions).

### Identification of candidate transcription-factor binding sites

We scanned for candidate binding sites in upstream fragments with FIMO [37], using positional weight matrices downloaded from PRODORIC [38]. Candidate binding sites were filtered using the FDR correction for multiple testing (*q*<0.05).

### Gene composition of the leading and lagging strands

We identified origin (*OriC*) and terminus (*Ter*) of replication analyzing GC-skew plots. Based on the *OriC* and *Ter* locations, we determined the strands for genes from different fractions of the pan-genome. To test the statistical significance of differences between the pan-genome fractions, we performed a permutation test by shuffling genes between pan-genome fractions (retaining the fractions sizes) 250 times, thus obtaining the distribution of differences between the fractions under the random null model, and compared the observed differences with this distribution. Calculated differences with *p*-value satisfying the threshold with the Bonferroni correction for multiple testing were considered as statistically significant.

Statistical significance of over-representation of inter-replichore inversions was calculated as the probability of a given number of inter-replichore inversions in the set of inversions with given lengths. The probability of occurrence of the origin or the terminator of replication within the inversion was calculated as the ratio of the inversion length to the replichore length.

### Construction of phylogenetic trees

For construction of phylogenetic trees we used concatenated aligned amino acid sequences of all core genes reverse translated to nucleotide alignment. Then maximum likelihood trees were constructed by RAxML [39] with default parameters.

### Synteny blocks and rearrangements history

Synteny blocks were constructed using the Sibelia algorithm [40] with default parameters for whole-genome nucleotide alignments. Blocks observed in a genome more than once were filtered out. The history of inversions was reconstructed using the MGRA algorithm [41].

### Detection of gene inflow

To detect genes horizontally transferred into species, we used the following model. If a gene with a mosaic phyletic pattern has been inherited vertically from the common ancestor and lost by several genomes, we expect to find it at the same syntenic region in the remaining strains. Genes not satisfying this condition are candidates for having been obtained horizontally. For this analysis, we excluded genes whose universal neighbours were affected by the reconstructed rearrangements, that is, genes located at or near boundaries of synteny blocks.

## Results and discussion

### Pan-genome and its fractions

We constructed 5742 OGs comprising 192782 genes. The number of genes in a genome assigned to OGs was 1872±178 with the median 1857 (Additional files 1: Tables S1 and 3: Table S2); the number of singletons was 48±53, median=22.

**Fig. 1:**
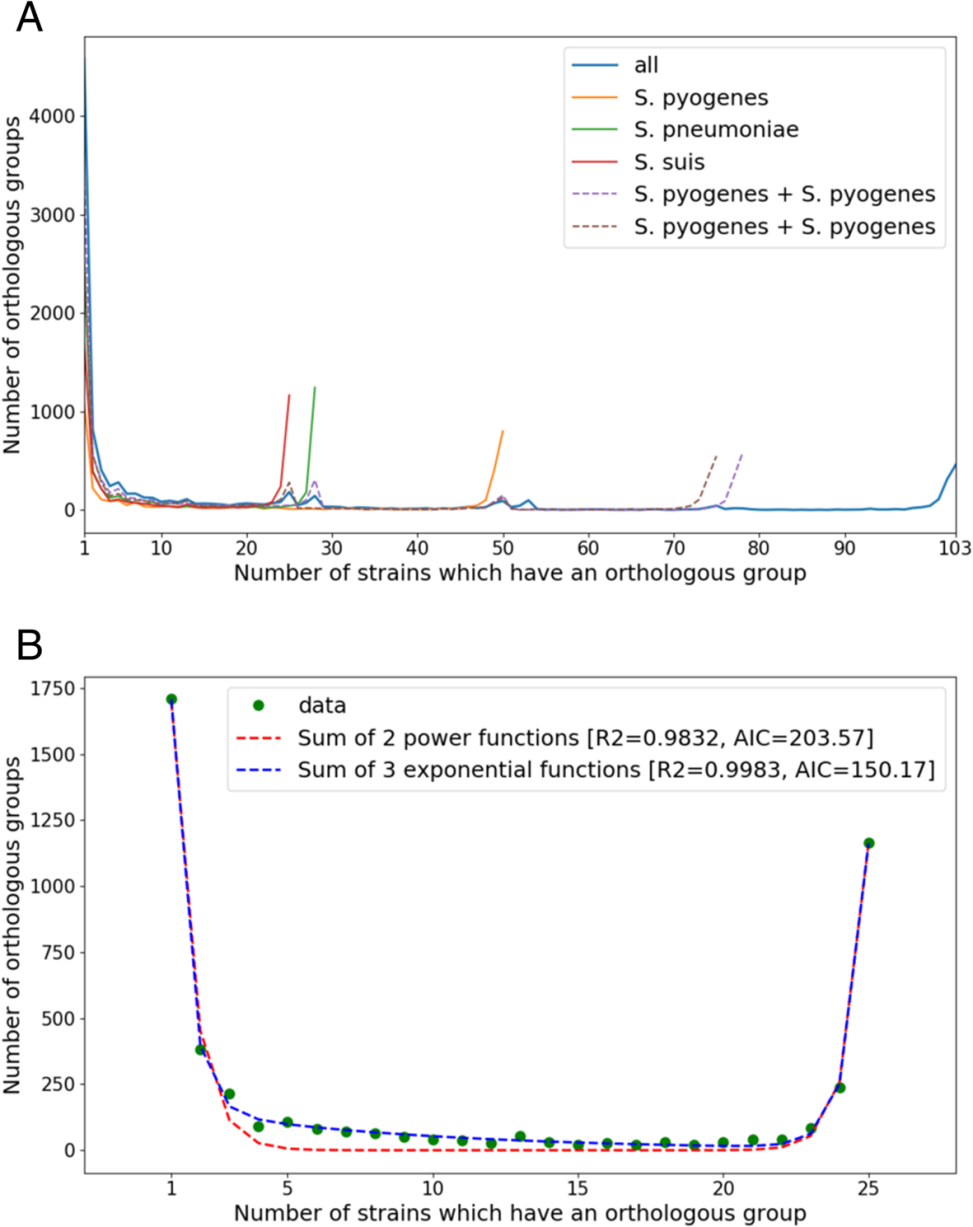
Distribution of orthologous groups (OGs) by the number of strains in which they are present. **a** For all analyzed strains (25 *S. suis*, 28 *S. pneumoniae*, and 50 *S. pyogenes*). **b** For 25 strains of *S. suis* with fitting by the sum of three exponential functions and by the sum of two power functions

For strains of each species and for combinations of species we performed the standard pan-genome analysis to characterize the distribution of OGs by strains and to estimate the sizes of core and pan-genomes. The distribution of OGs by strains had a typical U-shape [9, 10, 42] (Fig. 1a), that could be fitted by a sum of three exponents (as in [42]), describing the common core, periphery genes, and unique genes, or by a sum of two power law functions (as in [10]), that divide the pan-genome into almost universal and almost unique genes (Fig. 1b). These two fits had almost equal R-squared values, but based on the AIC, the sum of three exponents was slightly more significant. When all three species were combined, the U-curve had additional minor peaks reflecting species-specific genes (Fig. 1a).

**Fig. 2:**
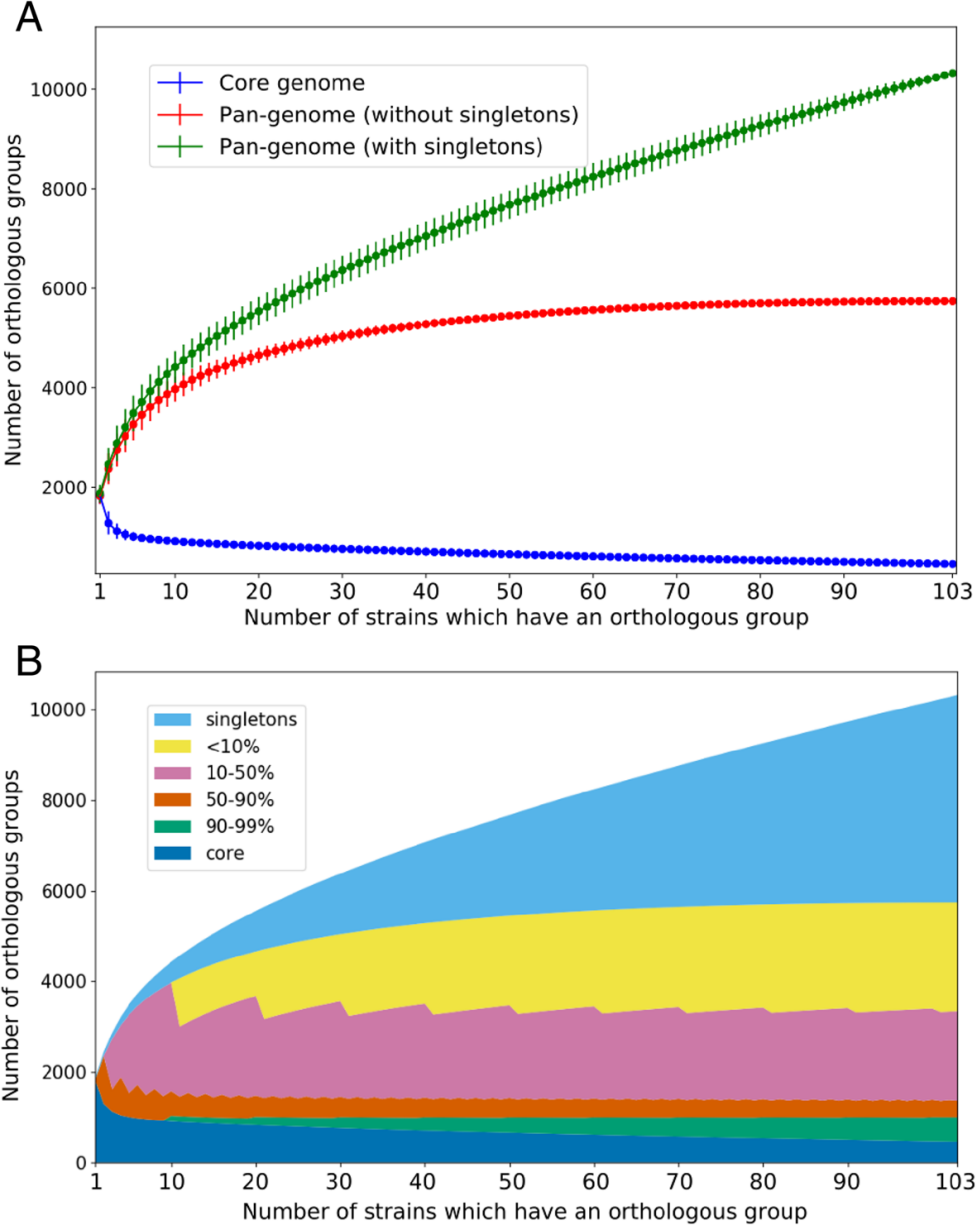
Sizes of the pan-genome fractions. **a** Sizes of the core genome, the pan-genome without singletons, and the total pan-genome with singletons as functions of the number of the analyzed strains. **b** Number of orthologous groups (OGs) are present in a given fraction of strains. Core OG present in all strains, singletons are present in only one strain each. All fractions reach saturation, while the pan-genome continues to grow due to singletons

The core genome of the three species converged to 458 genes (core and pan-genomes of the species are described in Table 1). The pan-genome was open, exceeding 10300 genes (Fig. 2a). Species-specific pan-genomes were also open, with core genomes accounting for approximately half of genes in any given genome. The Chao approximation of the total pan-genome size was 23217. The fraction of unique genes in a genome was less than 4%, with the highest fraction of unique genes in *S. pneumoniae*, and the lowest one in *S. pyogenes* (Additional file 4: Figure S2). The latter observation is partially explained by the presence of some very close strains in the analyzed genome set.

**Table 1:** Size of pan-genome fractions

Species	Core genes	>90*%*	>50*%*	>10*%*	Not specie-specific	Total pan-genome	Chao estimation
All	458	999	1361	3339	5742	10326	23217
*S. suis*	1164	1486	1803	2582	3264	4672	8562
*S. pneumoniae*	1243	1480	1838	2768	3410	5672	13680
*S. pyogenes*	800 (1017*)	1403	1588	2258	2843	3757	6477

* - without *S. pyogenes STAB901* and *S. pyogenes MTB313* which were excluded from the RefSeq database

As in [10], we split the pan-genome into percentile fractions by considering OGs present in at least a given fraction of strains. All such pan-genome fractions reach saturation after addition of the first few strains, an exception being the core genome, that continues shrinking, although at a decreasing rate, and the total pan-genome that grows, mostly due to strain-specific, unique genes. If unique genes are excluded, the total pan-genome becomes closed and converges to about 5750 genes (Fig. 2b and Table 1).

**Fig. 3:**
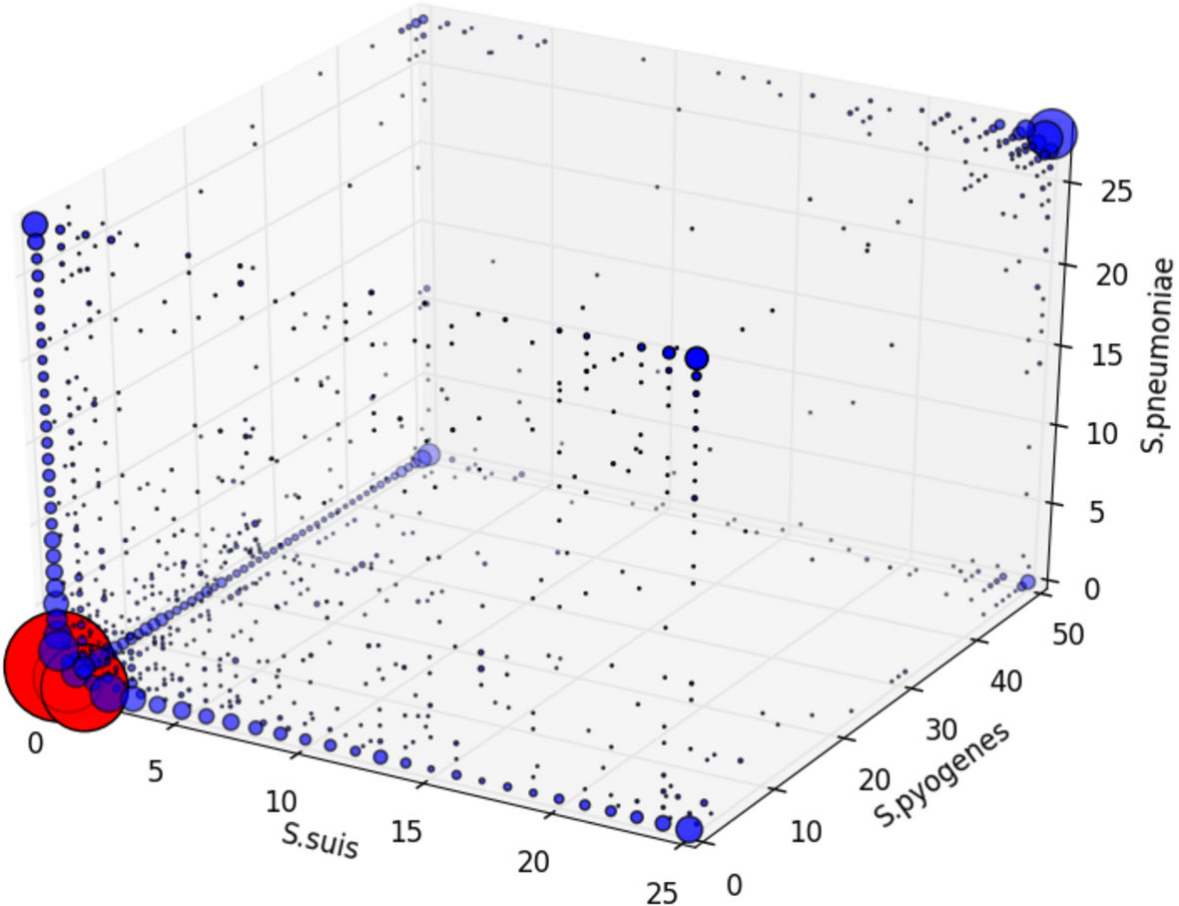
Distribution of orthologous groups (OGs) by the number of strains in which they are present as a plot in three dimensions. Axes correspond to species. Size of dots reflects the number of OGs. Red dots marks singletons OGs. Most dots reside at edges or in corners of the cube plot; Additional file 5: Figure S3 contains two-dimensional projections corresponding to pairwise comparisons

We also considered the OG distribution in all three species simultaneously as a plot in three dimensions (Fig. 3, Additional file 5: Figure S3). Excluding singletons, the largest group of OGs was formed by species-specific periphery (1136 in *S. pneumoniae*, 891 in *S. suis*, 922 in *S. pyogenes*), then OGs from the common core of the three species (458 OGs; or 825 OGs for a more relaxed definition with OG allowed to be absent in one strain in each species), then OGs belonging to the inner space of the plotted cube, i.e. to the common periphery of all three species (270 OGs), species-core OGs (114 in *S. pneumoniae*, 126 in *S. suis*, 87 in *S. pyogenes*), and, finally, some OGs formed common cores of species pairs to the exclusion of the third species (93 for *S. pneumoniae* and *S. suis*, 30 for *S. suis* and *S. pyogenes*, 12 for *S. pneumoniae* and *S. pyogenes*, reflecting closer phylogenetic relationships between *S. pneumoniae* and *S. suis*).

### Distribution of GO terms across pan-genome fractions

Interproscan [24] provided at least one GO term to 127672 genes. These assignments are largely consistent, as members of an orthologous group tend to be assigned the same GO term (Additional file 6: Figure S4). Requiring that at least 90% of proteins from an OG share the GO term, we assigned GO terms to 2969 orthologous groups.

**Fig. 4:**
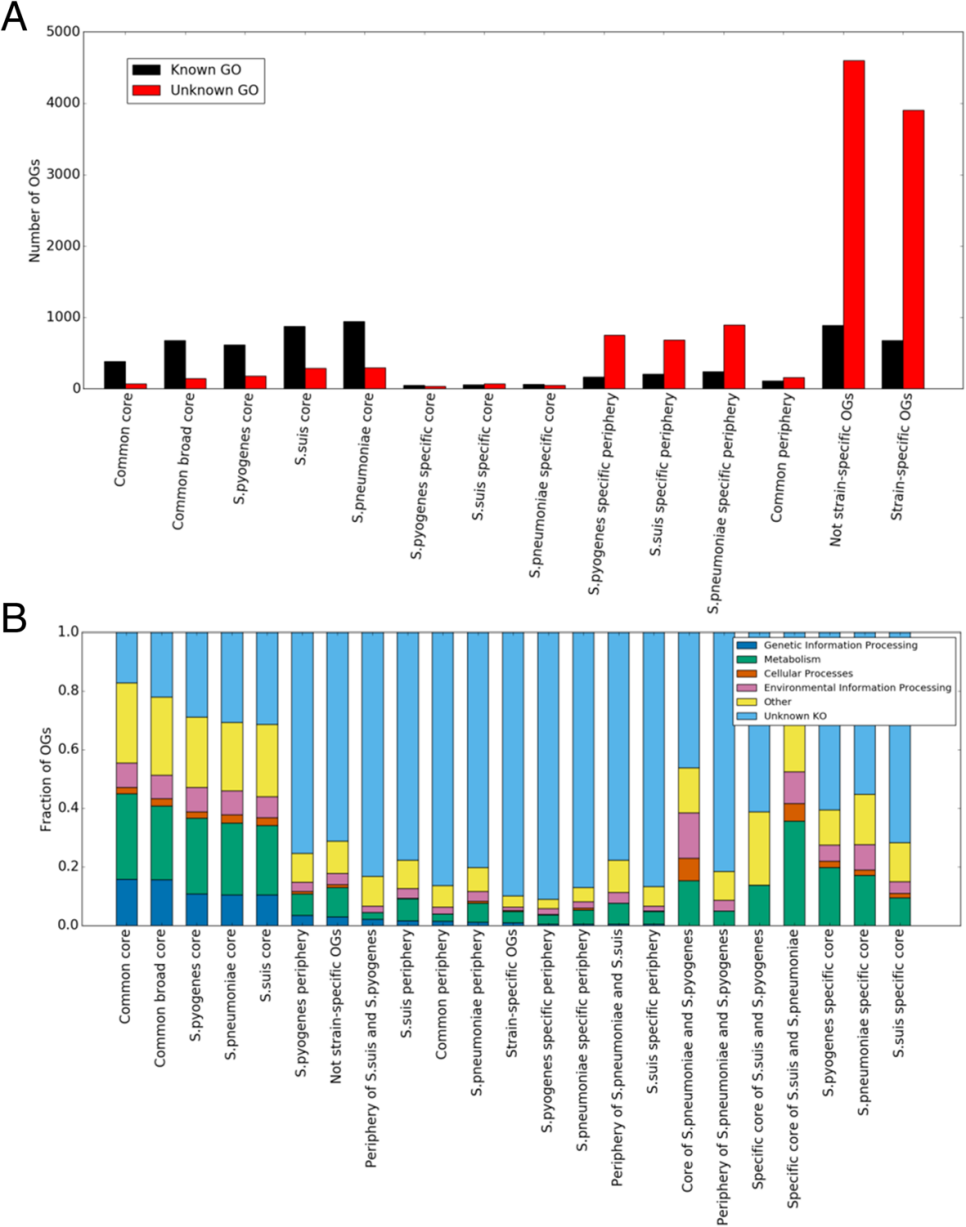
Distributions of orthologous groups (OGs) (**a**) with or without a GO term and (**b**) with known or unknown high-level KEEG KO category across the pan-genome fractions. Pan-genome fractions: Common core - OGs present in all strains, Common broad core - OGs missing at most in one strain of each species, *S. pyogenes* core - OGs present in all *S. pyogenes* strains, *S. suis* core - OGs present in all *S. suis* strains, *S. pneumoniae* core - OGs present in all *S. pneumoniae* strains, *S. pyogenes* specific core - OGs present in all *S. pyogenes* strains and absent in other species, *S. suis* specific core - OGs present in all *S. suis* strains and absent in other species, *S. pneumoniae* specific core - OGs present in all *S. pneumoniae* strains and absent in other species, *S. pyogenes* specific periphery - OGs present in some *S. pyogenes* strains and absent in other species, *S. suis* specific periphery - OGs present in some *S. suis* strains and absent in other species, *S. pneumoniae* specific periphery - OGs present in some *S. pneumoniae* strains and absent in other species, common periphery - OGs present in some but not all strains of each species, not strain-specific OGs - all OGs excluding singleton OGs, strain-specific OGs - singleton OGs, *S. pyogenes* periphery - OGs present in some *S. pyogenes* strains, periphery of *S. suis* and *S. pyogenes* - OGs present in some *S. suis* and *S. pyogenes* strains,*S. suis* periphery - OGs present in some *S. suis* strains, *S. pneumoniae* periphery - OGs present in some *S. pneumoniae* strains, periphery of *S. pneumoniae* and *S. suis* - OGs present in some *S. pneumoniae* and *S. suis* strains, specific core of *S. pneumoniae* and *S. pyogenes* - OGs present in all *S. pneumoniae* and *S. pyogenes* strains and absent in *S. suis* strains, periphery of *S. pneumoniae* and *S. pyogenes* - OGs present in some *S. pneumoniae* and *S. pyogenes* strains, specific core of *S. suis* and *S. pneumoniae* - OGs present in all *S. suis* and *S. pneumoniae* strains and absent in *S. pyogenes* strains, specific core of *S. suis* and *S. pyogenes* - OGs present in all *S. suis* and *S. pyogenes* strains and absent in *S. pneumoniae* strains

The distribution of orthologous groups with determined GO terms across the pan-genome, given in Fig. 4a, shows that core-genome groups tend to be more often assigned GO terms than genes from the unique fraction of the pan-genome. Indeed, the unique genes mainly had no GO terms (Fig. 4a, “Strain-specific OGs”) or KEGG KO terms (Fig. 4b, “Strain-specific OGs”, Additional file 7: Figure S5) and were annotated as “hypothetical proteins”, hence likely encoding mobile elements and phage-related proteins or simply resulting from genome misannotation (Additional file 8: Figure S6). However, some important gene groups also fell in this category, as 15% of unique genes were predicted to be virulence-related (Additional file 9: Figure S7). The exact fraction of functionally relevant genes in this group is hard to estimate, as the absence of homologs makes functional annotation almost impossible (although gene calling artifacts in some cases may be recognized by the comparison of strains).

Overrepresented functional categories in different fractions of the pan-genome with regards to the described cube representation are shown in Additional file 3: Table S2. The common core genome and weakly species-specific cores, that is genes observed in all strains of one species and some strains of the remaining species, are enriched with GOs involved in information processing, such as translation, ribosome, gene expression, RNA, and all kinds of metabolic processes. The periphery is enriched in a small set of functions, including response to other organisms and pathogenesis (this fraction features the highest percent of predicted virulence-related genes, Additional file 9: Figure S7), in particular, sialidase activity (*S. pyogenes*, *S. pneumonie*), DNA binding and some carbohydrate-related functions (*S. pneumoniae*, *S. suis*), as well as transcription factors (*S. pyogenes*). Strain-specific genes are mainly enriched in transposase activity, DNA recombination, and DNA integration, consistent with the origin of strain-specific genes from mobile elements [13]; in addition, these categories are enriched in orthologous groups from the common periphery, that is, among genes present in a fraction of strains from all three species. Species-specific cores are enriched in vitamin biosynthesis (*S. pneumonie*), transport, histidine and lactose metabolism, and response to oxidative stress (*S. pyogenes*), and iron transport, amino acid metabolic processes, and regulation of transcription (*S. suis*).

The distribution of KEGG KO categories across the pan-genome is shown in Fig. 4b. The fraction of orthologous groups assigned with a KO category decreases when moving from the core genome to the periphery and then to strain-specific genes. Most orthologous groups related to “Genetic Information Processing”, that can be considered as most essential groups, correspond to the common core, followed by the periphery and then strain-specific genes; no such orthologous groups were found among species-specific cores.

Hence, the functional distribution agrees with the pan-genome model in which the core is responsible for information and most metabolic processes, the periphery performs fine-tuning of bacteria to specific ecological niches, and strain-specific genome fraction is comprised mainly of mobile elements-related genes [43].

Genes from the common core show a weak preference to the leading strand, whereas periphery and strain-specific genes tend to be located at the lagging strand (Table 2). The leading strand preference of the core genes may be associated with their higher transcription level and/or with essentiality of these genes [44]. However, this difference is not very strong, as *Streptococcus* feature a general, strong bias with about 80% of genes located at the leading strand.

**Table 2:** Strand preference of genes from pan-genome fractions

Pan-genome fraction (number of OGs)	Percent of genes on leading strand	Standart deviation
All genes (5742 OGs + 4584 singletons)	79.6	1.4
Non strain-specific OGs (5742)	79.8 *	1.4
Common core (458)	83.9 *	0.6
*S.pneumoniae* specific core (114)	79.6	0.9
*S.pyogenes* specific core (87)	73.5 *	0.2
*S.suis* specific core (126)	78.8	1.6
*S.pneumoniae* periphery (1136)	75.2 *	5.2
*S.pyogenes* periphery (922)	75.5 *	3.6
*S.suis* periphery (891)	78.3 *	3.3
Common periphery (270)	77.8 *	6
Strain-specific OGs (4584)	70.1 *	15.2

Here, periphery is defined as genes present in some strains of a given species and absent in other species. * - statistically significant

### Selection regime in the pan-genome fractions

**Fig. 5:**
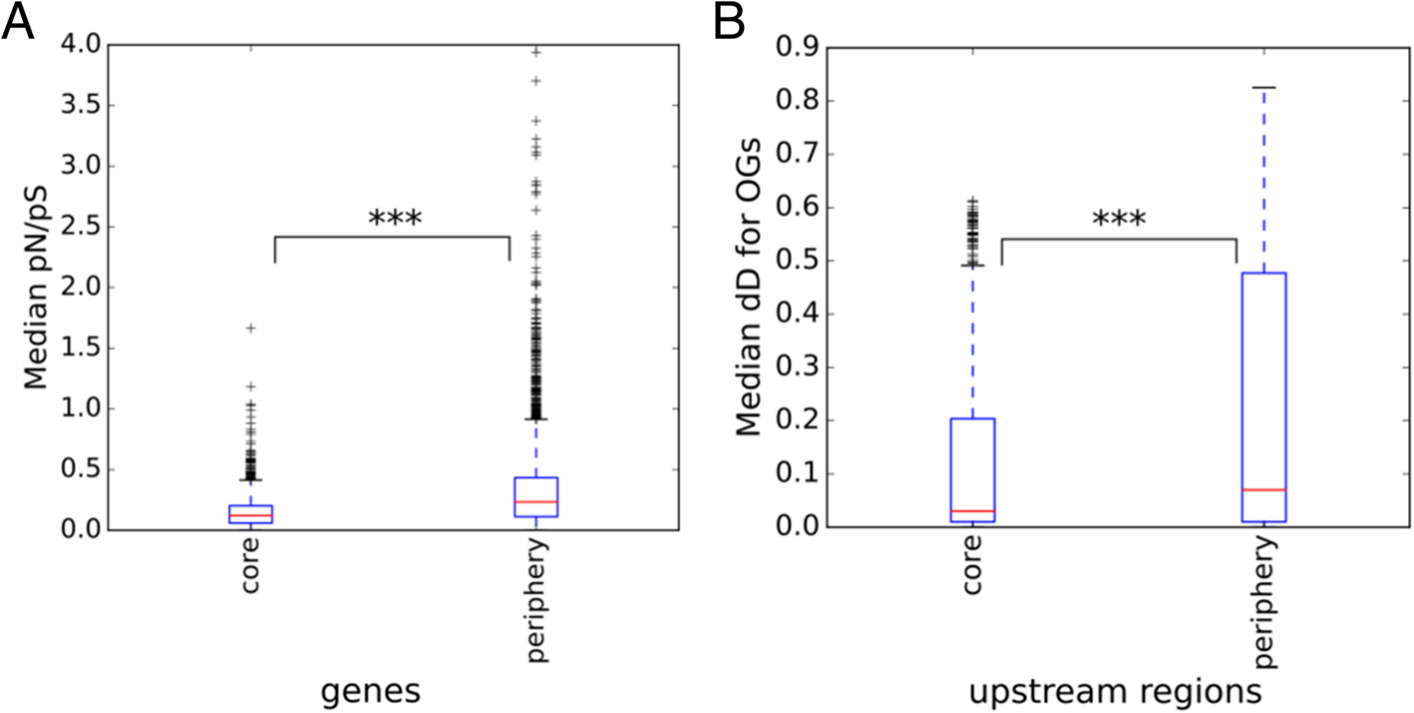
(**a**) Median value of the *pN*/*pS* ratio with the Jukes-Cantor correction for genes and (**b**) median number of nucleotide substitutions in upstream regions (*dD*). Significance level is indicated with stars (*p*-value <0.001)

Genes from the core genome encoding essential, housekeeping functions should evolve under higher purifying selection [45] yielding lower *pN*/*pS* ratio, compared to dispensable genes from the periphery genome. Indeed, as shown in Fig 5a and Additional file 10: Figure S8a, the *pN*/*pS* ratio is the smallest for the core genome (Mann–Whitney, *p* <0.01 for comparisons of core and periphery fractions within the same species).

In addition to protein-coding genes, purifying selection acts on regulatory elements in intergenic regions. In this and the next sections, we attempt to quantify this selection by determining the fraction of intergenic nucleotide positions evolving under negative selection and by comparing regions that are deleted in some strains with universal intergenic regions.

The median fraction of nucleotide substitutions (with the Jukes-Cantor correction) in intra-species alignments of orthologous upstream regions was 5.6%. The distribution of the number of nucleotide substitutions with the Jukes-Cantor correction, *dD*, is shown in Fig. 5b and Additional file 10: Figure S8b. The fraction of the pan-genome with the lowest number of substitutions is the core genome. Hence, not only the core genes, but their expression level and regulation are likely to be conserved.

In inter-species alignments, conserved columns may indicate functional conservation or simply insufficient time after speciation to accumulate mutations in all non-essential positions. To estimate the number of hidden non-conserved positions we used the method from [36]. We have calculated that only 10-20% of positions in the upstream regions evolve under purifying selection (Additional file 11: Figure S9). However, this may be an underestimate due to the large distance between the analysed species and the low number of conserved positions.

### Inserted and deleted fragments in intergenic regions are not neutral

**Fig. 6:**
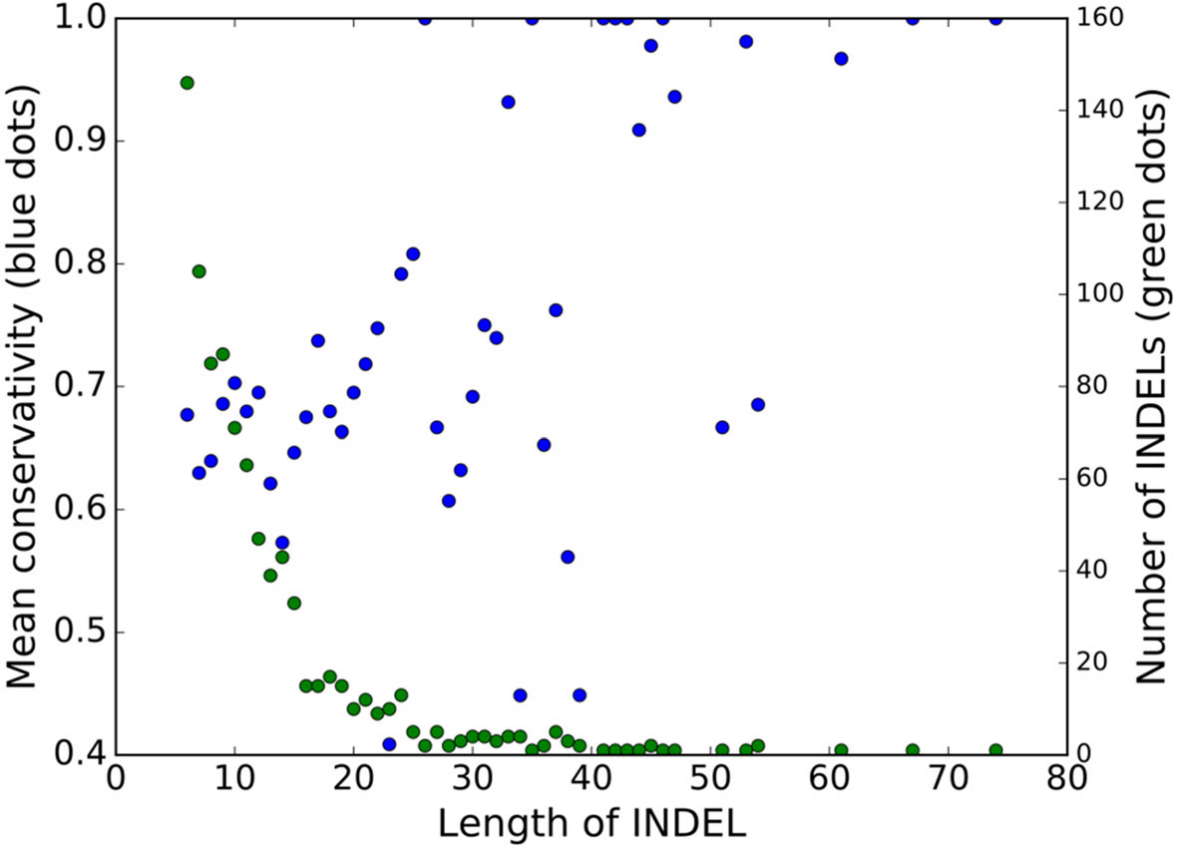
Dependence of the mean conservation level of nucleotides in indels in upstream regions in genome of *S. pyogenes* on the indel size. Blue dots correspond to the mean conservation level, green dots correspond to the number of indels of this size

Many alignments of orthologous upstream regions contained extended insertions and/or deletions (indels). One might expect that sequences within indels are selectively neutral. However, the indel fragments demonstrate strong sequence conservation (in the remaining genomes), and the level of conservation increases with the indel length (Fig. 6, Additional file 12: Figure S10). One possible explanation could be horizontal transfer of regulatory sequences enabling fast change of the level and mode of expression for the adjacent gene(s) [46]. However, computational scanning for candidate binding sites of transcription factors (see Methods) in the indel fragments has not produced an excess of candidate sites compared with control, random fragments from the same upstream regions, controlled for length. This might be due to low recall of the recognition rules, noise in predictions, and the fact that evolving intergenic regions may contain genes for regulatory RNAs [47, 48].

### Genomic rearrangements and antigenic variation of histidine triad protein PhtD

An important mode of genome evolution is rearrangements of chromosome fragments. In prokaryotes with single chromosomes the prevalent type of rearrangements are symmetrical inversions around the origin of replication [49–52]. While several inversions in some *Streptococcus* strains had been described [53], the increased phylogenetic coverage allowed us to actually map the events to the phylogenetic tree.

Synteny blocks were obtained using whole-genome alignments for each species. Only blocks present in all strains were used for the reconstruction of inversions. As a result, 13 inversions for *S. pneumoniae*, 21 inversions for *S. suis*, and 26 inversions for *S. pyogenes* were identified. Mapping these events to phylogenetic trees (Additional file 13: Figure S11) revealed cases of parallel inversions in all three species.

The observed parallel inversions could be explained by homologous recombination (horizontal transfer between strains) involving a segment containing the inverted fragments. If this were the case, sequence trees constructed using the genes from the inverted fragments would cluster together strains with the parallel inversions. However, such trees for all inversions are consistent (Additional file 14: Figure S12) with the benchmark tree constructed using the alignments of all core gene (Additional file 2: Figure S1) confirming the independent origin of these inversions.

Previously, inversions in *Streptococcus* spp. were explained by selection to rebalance the replichore architecture affected by insertion of prophages [14]. To check this hypothesis, we compared lengths of prophage regions in strains that contained the same inversion, and vice versa the number of inversions in strains with the same rate of prophage insertions (Additional file 1: Table S1). No correlation between the rates of prophage insertions and inversions was observed.

**Fig. 7:**
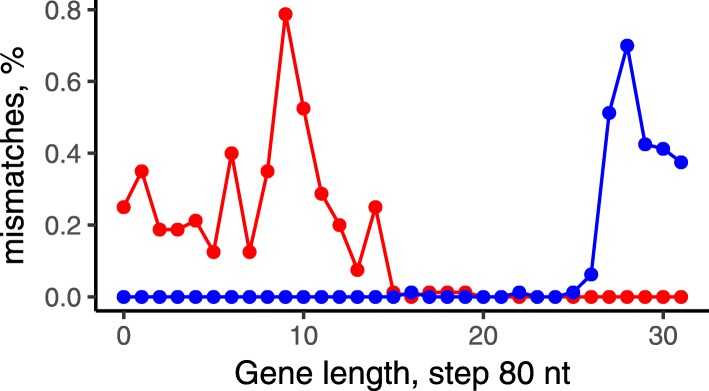
Number of mismatches in alignments of the histidine-triad proteins in *S. pneumoniae* ST556 and *S. pneumoniae* TCH8431/19A. The red points and blue points show the local dissimilarity level of PhtB from *S. pneumoniae* ST556 vs PhtB in S. pneumoniae TCH8431/19A and PhtD from *S. pneumoniae* TCH8431/19A, respectively

All inversions were bounded by mobile elements or clusters of rRNA except one event in the *S. pneumoniae* subtree. This inversion of length 15 kB was found in four separate branches and breakpoints were formed by genes encoding the surface antigen proteins PhtB and PhtD from a family characterized by the presence of several histidine triad (HxxHxH) motifs. PhtD and PhtB are relatively large proteins with lengths about 850 amino acids thought to be involved in multiple functions, including metal ion homeostasis, evasion of complement deposition, and adherence of bacteria to host cells [54]. In pairs of strains with and without the inversion, these proteins are composed of two independent parts with the inversion breakpoint in the middle of the gene that might indicate the action of an antigen variation mechanisms (Fig. 7).

**Fig. 8:**
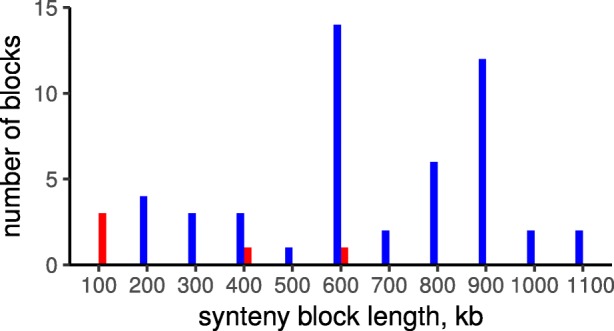
Distribution of inversion lengths. Red color corresponds to intra-replichore inversions; blue color, inter-replichore inversions

As more than 80% core genes in *Streptococcus* spp. are found on the leading strand (see above), one would expect strong selection against intra-replichore inversions, as they do switch genes between leading/lading strands. Indeed, inter-replichore inversions are overrepresented (57 events of 62, the *p*-value = 9×10^−14^) (Fig. 8).

### Detection of gene inflow

**Fig. 9:**
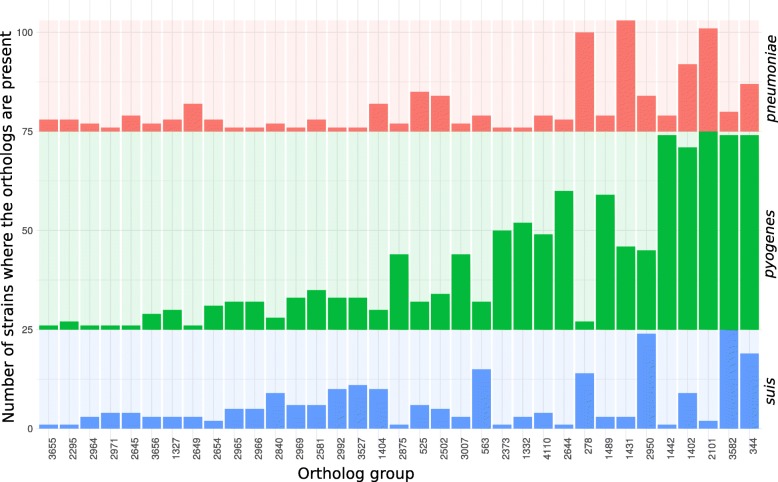
Distribution of genes with non-conserved genome positions and non-consistent phylogenetic trees. One column corresponds to one ortholog group. Bars show the fraction of genomes where the orthologs are present

To identify genes horizontally transferred after the divergence of the studied *Streptococcus* spp. and further spreading between the strains, we selected genes that were not unique and that were not common for at least one *Streptococcus* species (referred to periphery genes). Positions of single-copy, universal genes were analyzed to construct syntenic regions for all strains and to compile a set of periphery genes occurring in different syntenic regions and, therefore, likely being spread by horizontal gene transfer. The set comprised 277 orthologous groups that is about 7% of single-copy periphery genes. To confirm horizontal gene transfer, we constructed phylogenetic gene trees for all orthologous groups that were present at least in two *Streptococcus* species and at least in two strains in each species and checked whether each species were monophyletic, that is, formed a separate branch in these trees (Table 3). Most groups (88%) yielded trees with monophyletic species (consistent trees) and had conserved location in all genomes, indicating vertical inheritance from a common ancestor. About a half of groups with inconsistent trees had conserved genome positions that may be explained by homologous recombination; the remaining half had non-conserved location. The set of 48 orthologous groups with inconsistent trees and non-conserved positions are candidates for horizontal transfer between species. More than a half of these genes are rare in all three *Streptococcus* species; others are rare in at least one species (Fig. 9).

**Table 3:** Statistics of periphery genes

	Conserved location	Non-conserved location	All
Consistent tree	1181	128	1309
Inconsistent tree	44	48	92
All	1225	176	1401

An analysis of overrepresented GO terms in the set of orthologous groups with non-conserved positions, compared with all non-core groups (Table 4), yielded many functions involved in interaction with DNA (DNA binding, nucleic acid binding, sequence-specific DNA binding), that may be linked to regulation, but also to mobile elements (DNA integration). Other overrepresented functions such as ATP hydrolysis coupled proton transport, energy coupled proton transport against electrochemical gradient, proton-transporting V-type ATPase complex, etc., all are likely linked to the V-type ATPase, that is thought to be horizontally transfered from archaea [55].

**Table 4:** Overrepresented GO terms in genes with non-conserved location, compared with all non-core genes

GO term	Genes with non-conserved location	Total number of genes with this GO term	*P*-value	Description
GO:0003677	51	603	1.5×10^−3^	DNA binding
GO:0015991	4	5	2.2×10^−3^	ATP hydrolysis coupled proton transport
GO:0015988	4	5	2.2×10^−3^	Energy coupled proton transport, against electrochemical gradient
GO:0003676	58	762	3.3×10^−3^	Nucleic acid binding
GO:0016469	4	6	3.8×10^−3^	Proton-transporting two-sector ATPase complex
GO:0006818	4	7	6.2×10^−3^	Hydrogen transport
GO:0015992	4	7	6.2×10^−3^	Proton transport
GO:0043565	14	98	6.8×10^−3^	Sequence-specific DNA binding
GO:0015074	13	100	2.6×10^−2^	DNA integration
GO:0033176	2	2	5.5×10^−2^	Proton-transporting V-type ATPase complex
GO:0033179	2	2	5.5×10^−2^	Proton-transporting V-type ATPase, *V*_0_ domain

## Discussion

The pan-genome of many bacterial species including *Streptococcus* was shown to be open [56, 57]. In agreement with previous observations, the pan-genome of studied *Streptococcus* species is also open but it is mainly due to strain-specific genes. The pan-genome size exceeds 10300 genes, but if unique genes are excluded, the total pan-genome becomes closed and converges to about 5750 genes. Splitting the pan-genome into percentile fractions by considering OGs present in at least a given fraction of strains revealed the saturation of all such fractions after addition of the first few strains except the core genome and unique genes.

In a typical genome for studied *Streptococcus* species, one quarter of genes belong to the genus core genome; one quarter, to the species-specific core; most other genes are periphery ones, and a minority are strain-specific. The core genome of studied *Streptococcus* species is enriched with information-process and main metabolic functions and depleted with mobile elements and phage-related genes; the periphery fraction is enriched with niche-specific metabolic functions, including pathogenesis-related ones; and strain-specific genes are enriched with hypothetical genes and mobile elements, but also contain many virulence-related genes. At that, *Streptococcus* has a broad periphery and a huge repertoire of strain-specific genes. A large periphery fraction of pan-genome is thought to be a characteristic of organisms with large long-term effective population sizes and an ability to fill a variety of new niches [58].

Variation of selection regimes for genes and their upstream regions is consistent with the suggested evolutionary role of pan-genome fractions [12, 59]. Specifically, the core genes demonstrate a lower level of substitutions than periphery and unique genes and this tendency holds both for protein-coding sequences and for upstream regions [60–62]. More generally, while it is known that intergenic regions in bacteria experience purifying selection [63, 64], its strength appears to be different between pan-genome fractions. The fact that upstream regions of core genes have fewer substitutions might reflect stronger conservation of their regulation or more complex regulation, yielding a larger density of transcription-factor binding sites and other regulatory structures. On the other hand, fragments of intergenic regions that are deleted (or inserted) in some strains, are not less conserved than the surrounding regions, which might be a sign of newly evolving regulatory interactions or of ‘horizontal regulatory transfer’ [46, 65]. Evolution of intergenic regions in prokaryotes is a sparsely studied area, and new tools such as PIGGY [66] should accelerate the progress in this direction, specifically, by allowing for rapid analysis of additional, diverse species and genera.

In many bacteria, including *Streptococcus*, within-replichore inversions, that is, inversions with endpoints in the same replichore, have been shown to be relatively rare and significantly shorter than inter-replichore inversions [67–71]. Both non-random mutational processes and selection have been suggested as potential drivers of biased inversion landscapes [67, 69, 72, 73]. In more recent papers it was shown that symmetric inversion bias is not a universal feature of prokaryotic genome evolution but varies considerably across clades and the magnitude of the symmetric inversion bias is associated with various features of adaptive genome architecture, including the distance of essential genes to the origin of replication and the preferential localization of genes on the leading strand [74].

The pattern of inversions reconstructed in the studied *Streptococcus* species revealed a strong selection against intra-replichore inversions that, in agreement with previous observations, might be caused by strong preferential localization of genes on the leading strand (more than 80% of core genes). Despite low frequency of inversions, parallel inversions were observed in all three studied species. Most inversions were bound by mobile elements or clusters of rRNA, so most parallel events were likely to be caused by intragenome recombination linked to a limited number of repeated elements. The exception was the inversion in the *S. pneumoniae* subtree with breakpoints formed by genes encoding surface antigen proteins phtB and phtD. As the inversion was shown to exchange gene fragments, it is likely to indicate the action of antigen variation.

Phase variation is known to be an important mechanism that leads to phenotype diversification via intra-genomic recombination. Antigenic variation via inversions of short genomic fragments was shown to play a significant role for the *S. pneumoniae* infection influencing its pathogenicity [75]. While this paper was under review, antigen variation by the observed large parallel inversion between the *phtD* and *phtB* genes in *S. pneumoniae* had been confirmed [76]. The practical relevance of this observation comes from the fact that this protein is a candidate for a next-generation *pneumococcal* vaccine [77]. This shows that evolutionary and functional analysis of predicted parallel rearrangements with direct confirmation of this mechanism may identify possible cases of phase variation by inversions in human pathogens.

In the studied *Streptococcus* species, about 7% single-copy periphery genes occur in multiple syntenic regions. The genes with inconsistent trees and non-conserved genome position are rare in at least one species and have likely experienced horizontal transfer between species. Hence, a large periphery in the *Streptococcus* pan-genome is likely to be explained by horizontal gene transfer, that is known to be one of the major drivers of genome evolution [78, 79]. Horizontal gene transfer in *Streptococcus* is facilitated by the competence system and is associated with immune system [80]. Moreover, the early proof that DNA carries genetic information was provided by experiments with *pneumococcus* [81, 82]. This emphasizes the importance of pan-genome studies of medically relevant bacteria, as their pathogenicity may be affected by rare periphery or even strain-specific genes.

## Conclusions

Members of the genus *Streptococcus* have a highly dynamic, open pan-genome, that potentially confers them with the ability to adapt to changing environmental conditions, i.e. antibiotic resistance or transmission between different hosts. *Streptococcus* genome plasticity is shaped by a dynamic interaction of major evolutionary forces such as horizontal gene transfer, genome rearrangements, and propagation of mobile elements reflecting the ecological niche and the lifestyle. Hence, integrated analysis of all aspects of genome evolution is important for the identification of potential pathogens and design of drugs and vaccines.

## Additional files

Additional file 1 Supplementary file **Table S1**. List of analyzed *Streptococcus* strains. (XLS 21 kb)

Additional file 2 Supplementary file **Figure S1**. Phylogenetic tree of analyzed *Streptococcus* strains based on alignments of universal single-copied genes. (PDF 347 kb)

Additional file 3 Supplementary file **Table S2**. Overrepresented functional categories in different fractions of the pan-genome. (XLS 87 kb)

Additional file 4 Supplementary file **Figure S2**. Distribution of the number of singletons in strains belong to different species. (PDF 34 kb)

Additional file 5 Supplementary file **Figure S3**. Two-dimensional projections for the distribution of orthologous groups (Fig. 3) corresponding to pairwise comparisons. (A) *S. pneumoniae* - *S. suis*, (B) *S. pyogenes* - *S. suis*, (C) *S. pneumoniae* - *S. pyogenes*. Axes correspond to species. Size of dots reflects the number of OGs. Red dots marks singletons OGs. (PDF 70 kb)

Additional file 6 Supplementary file **Figure S4**. Dependence of the number of orthologous groups (OGs) assigned a GO term on the threshold for GO term assignment. The threshold is the minimal fraction of genes from an ortologous group that have a GO term. Singleton are not considered. (PDF 15 kb)

Additional file 7 Supplementary file **Figure S5**. Distribution of high-level KEGG KO categories across pan-genome fractions. (A) absolute values, (B) relative values of four major KO categories. Pan-genome fractions are defined as in Fig. 4. (PDF 560 kb)

Additional file 8 Supplementary file **Figure S6**. Proportion of orthologous groups with hypothetical or mobile/phage related genes in (A) strain-specific OGs and in (B) not strain-specific OGs. (PDF 74 kb)

Additional file 9 Supplementary file **Figure S7**. Distribution of orthologous groups with virulence-related genes across pan-genome fractions. Number of OGs in each pan-genome fraction is shown in brackets. Pan-genome fractions are defined as in Fig. 4. (PDF 139 kb)

Additional file 10 Supplementary file **Figure S8**. Distributions of (A) the median value of the *pN*/*pS* ratio with the Jukes-Cantor correction for genes and (B) median number of nucleotide substitutions in upstream regions (*dD*) of genes from OGs from different pan-genome fractions. The number of analyzed OGs from each pan-genome fraction is shown in brackets. The pan-genome fractions are defined as in Fig. 4. (PDF 1248 kb)

Additional file 11 Supplementary file **Figure S9**. Fractions of nucleotides under purifying selection in upstream fragments of core OGs as a function of the number of compared strains in pair-wise analysis of species. (PDF 17 kb)

Additional file 12 Supplementary file **Figure S10**. Dependence of the mean conservation level of nucleotides in indels on the indel size. Blue dots correspond to the mean conservation level, green dots correspond to the number of indels of this size. (A) *S. pneumoniae*, (B) *S. suis*, and (C) *S. pyogenes*. (PDF 504 kb)

Additional file 13 Supplementary file **Figure S11**. Phylogenetic trees based on genes involved in parallel inversions. (PDF 867 kb)

Additional file 14 Supplementary file **Figure S12**. Phylogenetic trees for *S. pneumoniae* (A), *S. suis* (B), *S. pyogenes* (C) based on the alignments of universal single-copy genes. The numbers at tree branches show the numbers of inversions. Strains with parallel inversions are marked by color labels. Strains with the same inversion are marked by the same color. (PDF 1279 kb)

Additional file 15 Supplementary file **Table S3**. Orthologous groups compositions. (CSV 12,628 kb)
