# Supplementary figures and images for: Micro-evolution of three Streptococcus species: selection, antigenic variation, and horizontal gene inflow
Source: BMC Evol Biol. 2019 Mar 27;19:83. doi: 10.1186/s12862-019-1403-6 (PMC6437910; doi:10.1186/s12862-019-1403-6)

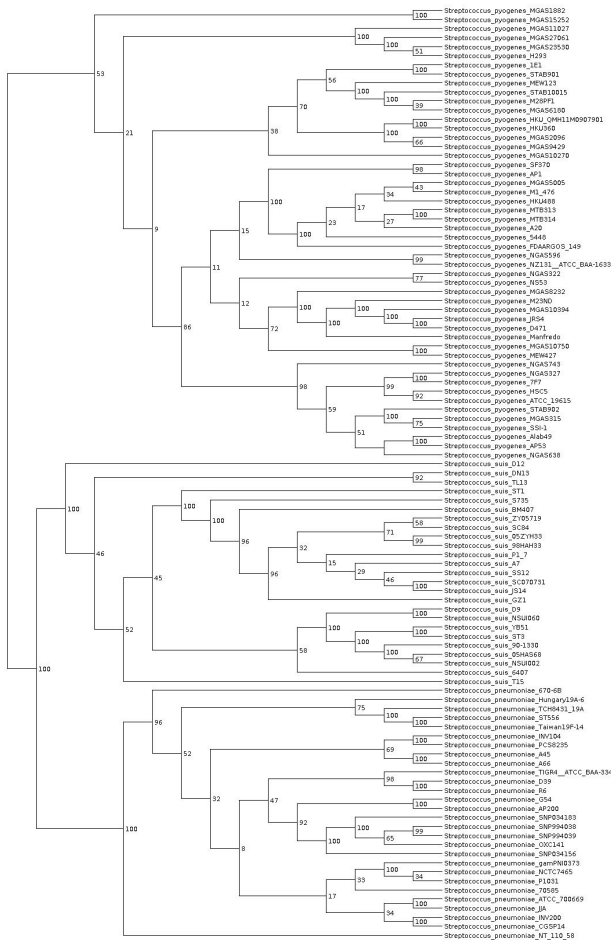

Supplement: Supplementary file 2 — Supplementary file Figure S1. Phylogenetic tree of analyzed Streptococcus strains based on alignments of universal single-copied genes. (PDF 347 kb) [file 12862_2019_1403_MOESM2_ESM.pdf]

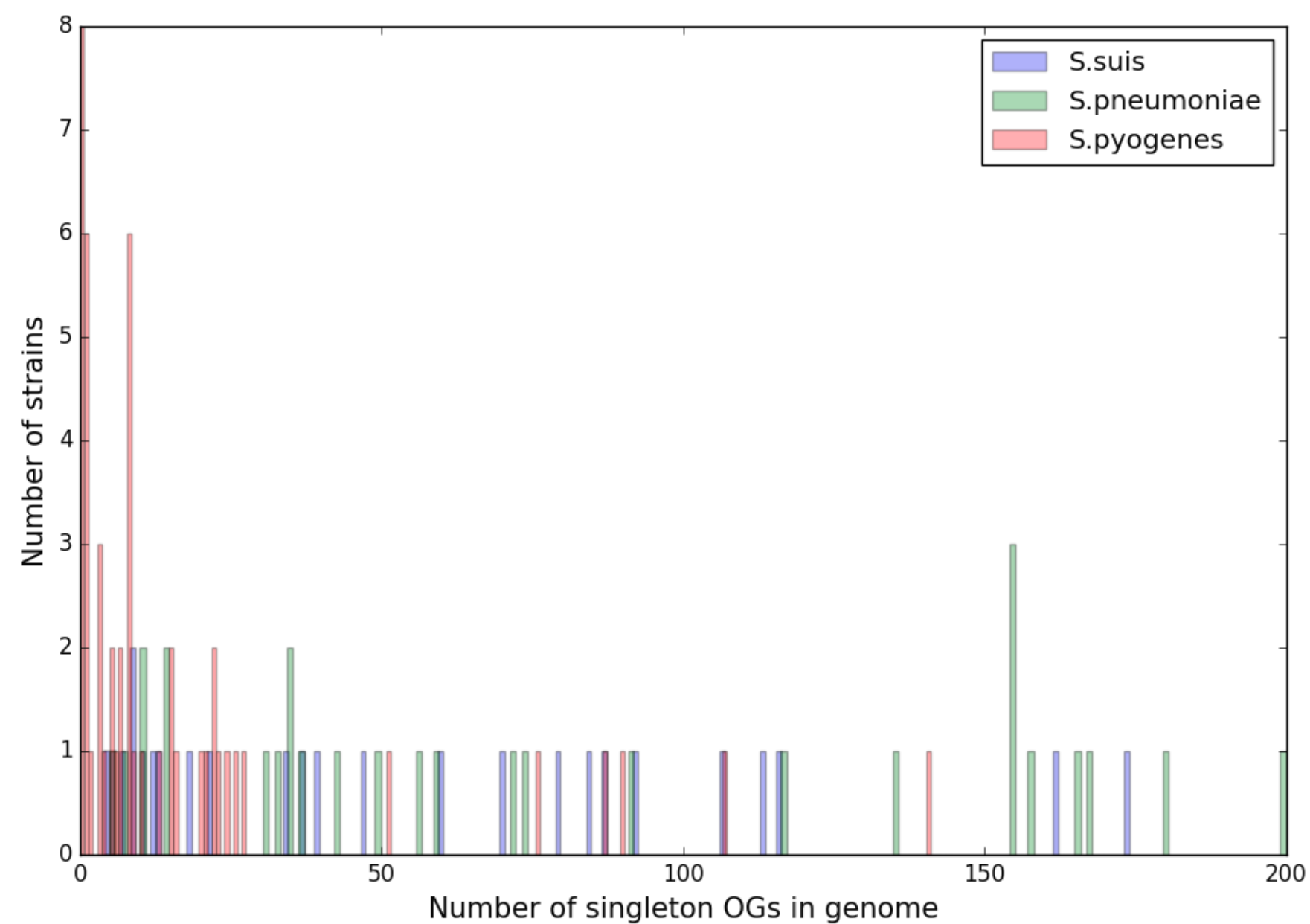

Supplement: Supplementary file 4 — Supplementary file Figure S2. Distribution of the number of singletons in strains belong to different species. (PDF 34 kb) [file 12862_2019_1403_MOESM4_ESM.pdf]

A

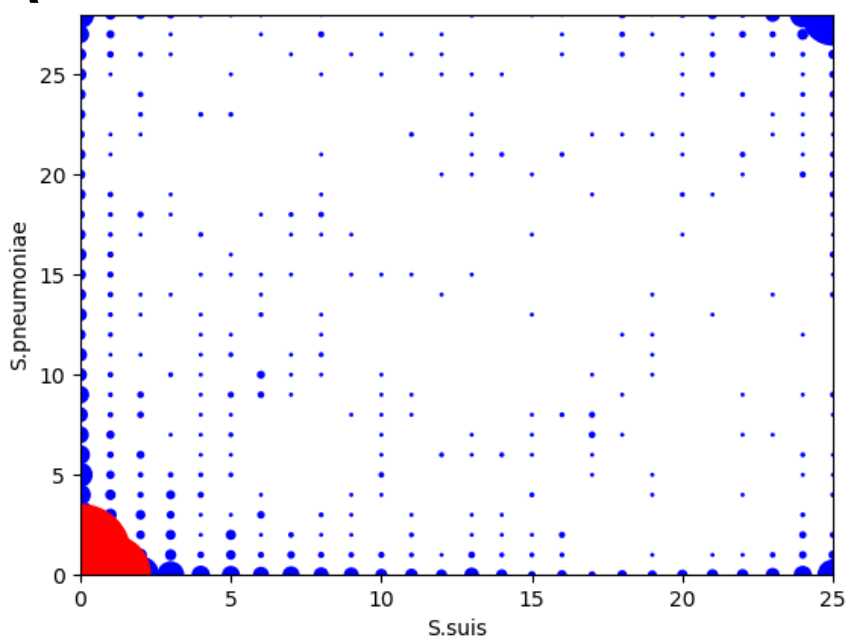

B

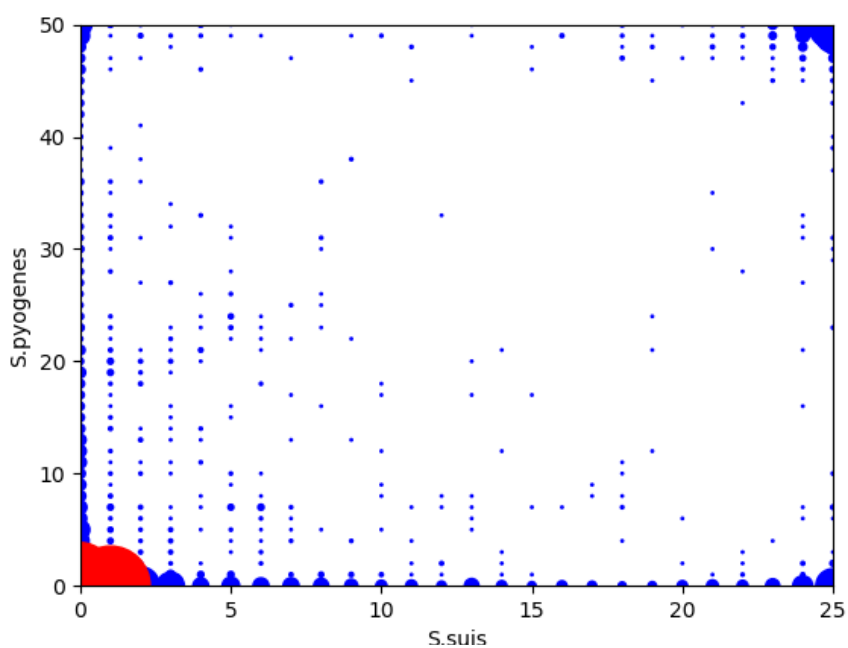

C

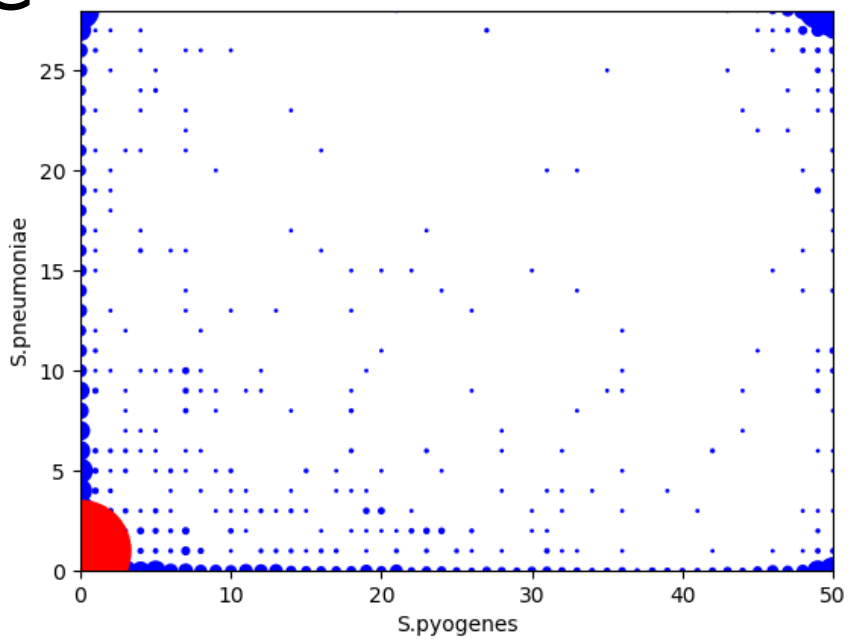

Supplement: Supplementary file 5 — Supplementary file Figure S3. Two-dimensional projections for the distribution of orthologous groups (Fig. 3) corresponding to pairwise comparisons. (A) S. pneumoniae - S. suis, (B) S. pyogenes - S. suis, (C) S. pneumoniae - S. pyogenes. Axes correspond to species. Size of dots reflects the number of OGs. Red dots marks singletons OGs. (PDF 70 kb) [file 12862_2019_1403_MOESM5_ESM.pdf]

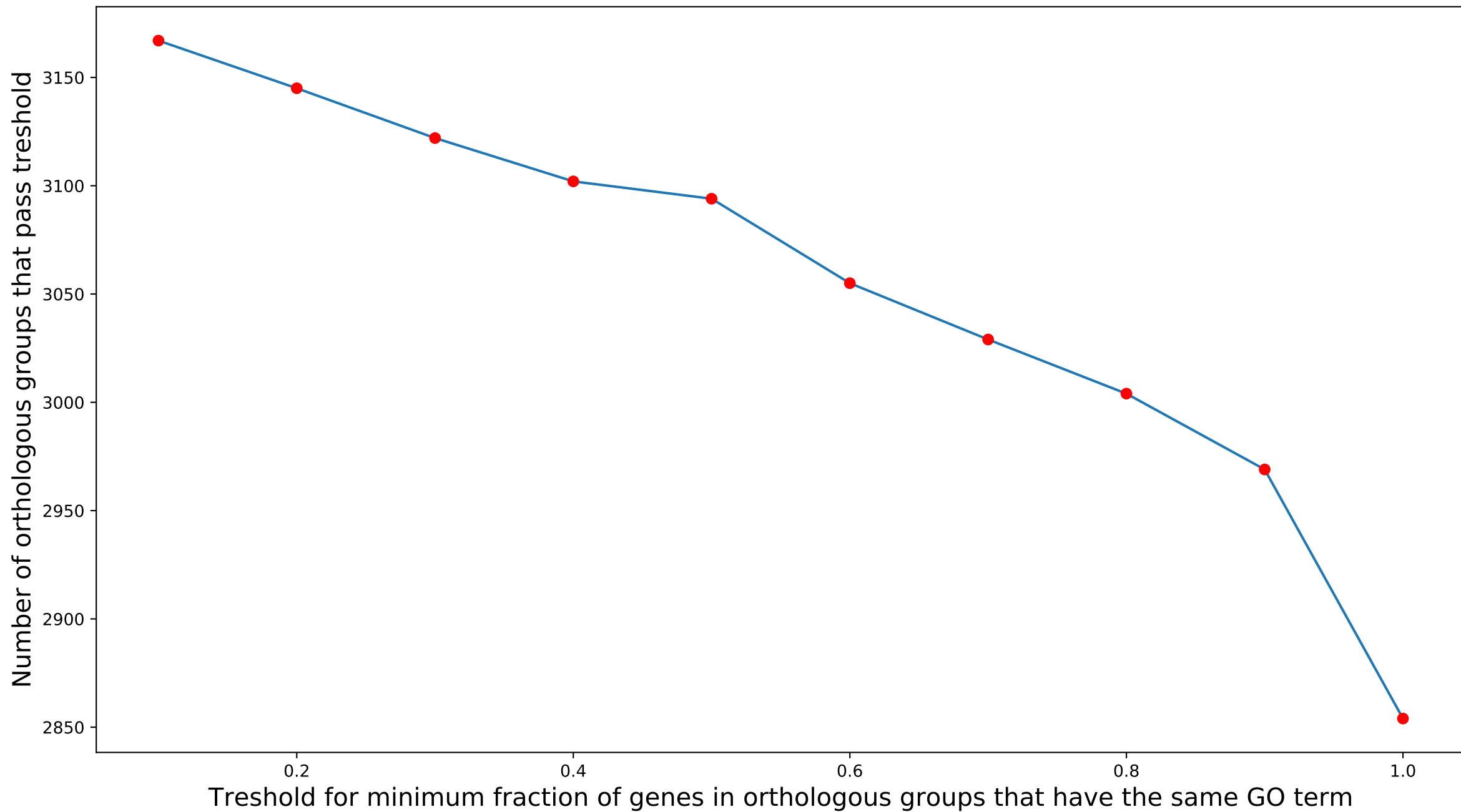

Supplement: Supplementary file 6 — Supplementary file Figure S4. Dependence of the number of orthologous groups (OGs) assigned a GO term on the threshold for GO term assignment. The threshold is the minimal fraction of genes from an ortologous group that have a GO term. Singleton are not considered. (PDF 15 kb) [file 12862_2019_1403_MOESM6_ESM.pdf]

A

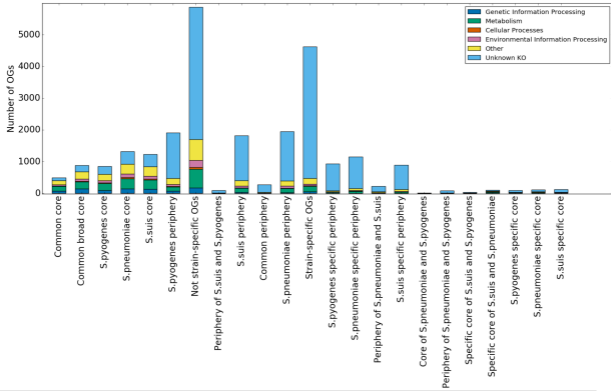

B

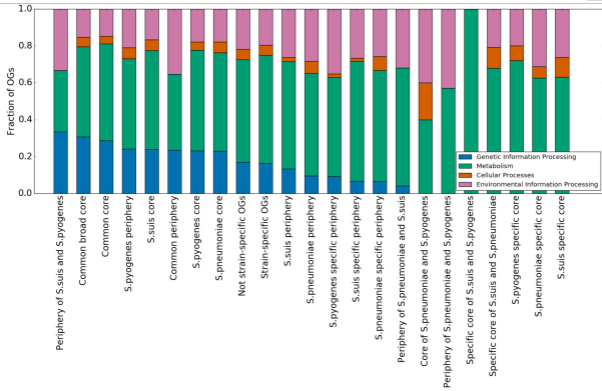

Supplement: Supplementary file 7 — Supplementary file Figure S5. Distribution of high-level KEGG KO categories across pan-genome fractions. (A) absolute values, (B) relative values of four major KO categories. Pan-genome fractions are defined as in Fig. 4. (PDF 560 kb) [file 12862_2019_1403_MOESM7_ESM.pdf]

(A) Strain-specific OGs

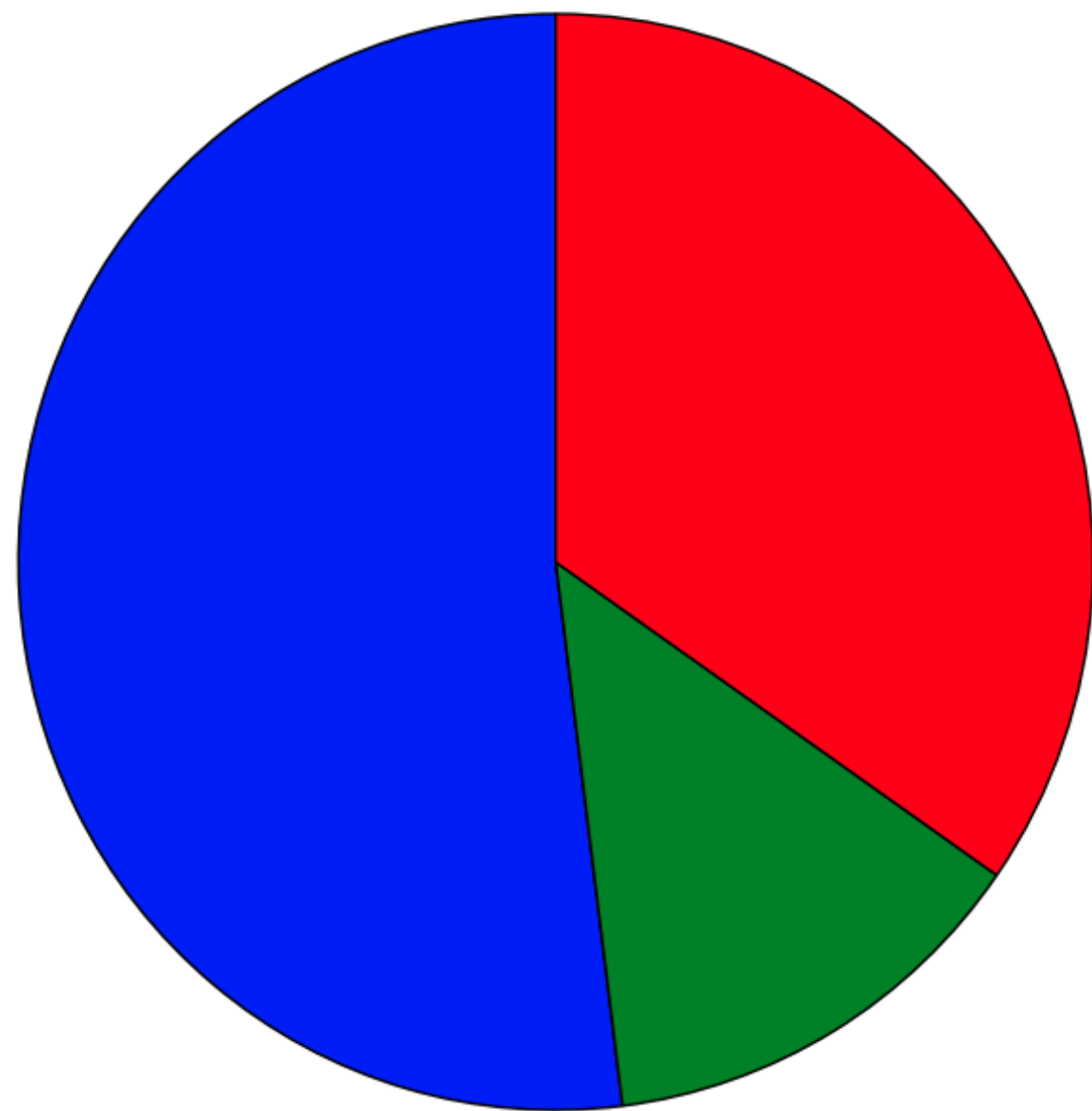

(B) Not strain-specific OGs

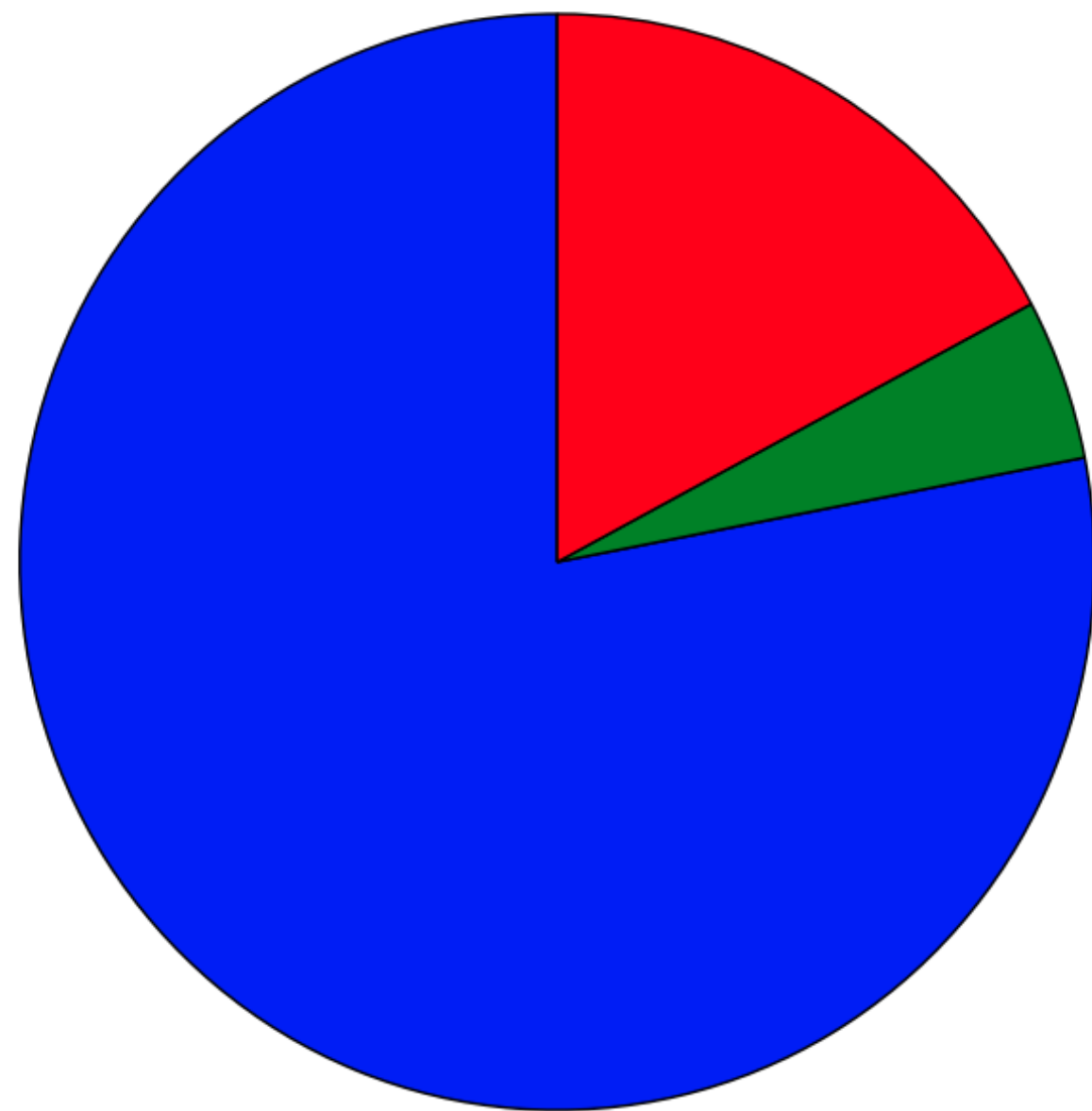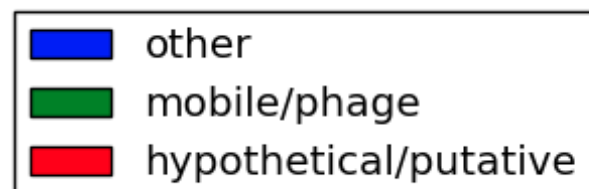

Supplement: Supplementary file 8 — Supplementary file Figure S6. Proportion of orthologous groups with hypothetical or mobile/phage related genes in (A) strain-specific OGs and in (B) not strain-specific OGs. (PDF 74 kb) [file 12862_2019_1403_MOESM8_ESM.pdf]

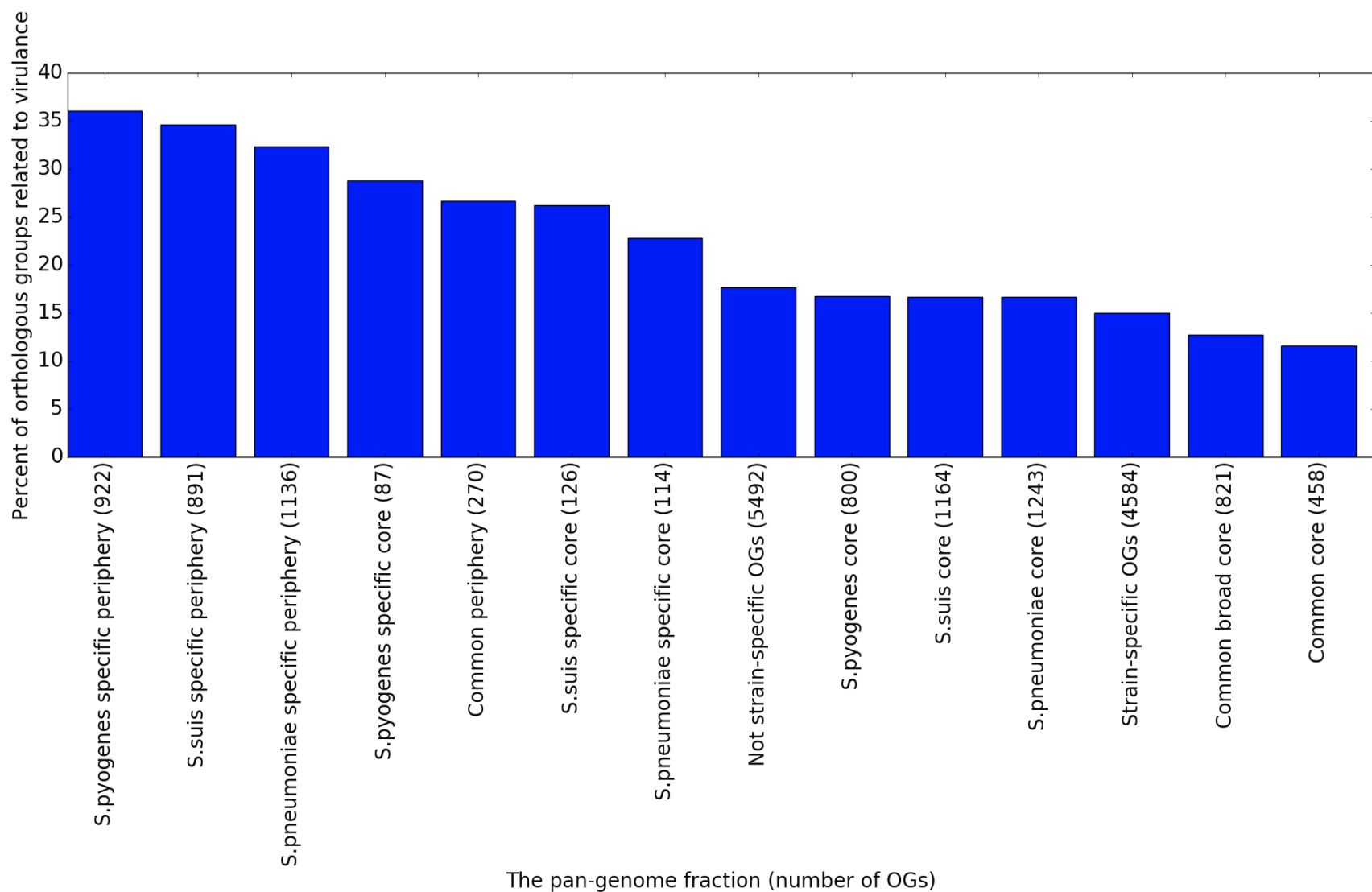

Supplement: Supplementary file 9 — Supplementary file Figure S7. Distribution of orthologous groups with virulence-related genes across pan-genome fractions. Number of OGs in each pan-genome fraction is shown in brackets. Pan-genome fractions are defined as in Fig. 4. (PDF 139 kb) [file 12862_2019_1403_MOESM9_ESM.pdf]

A

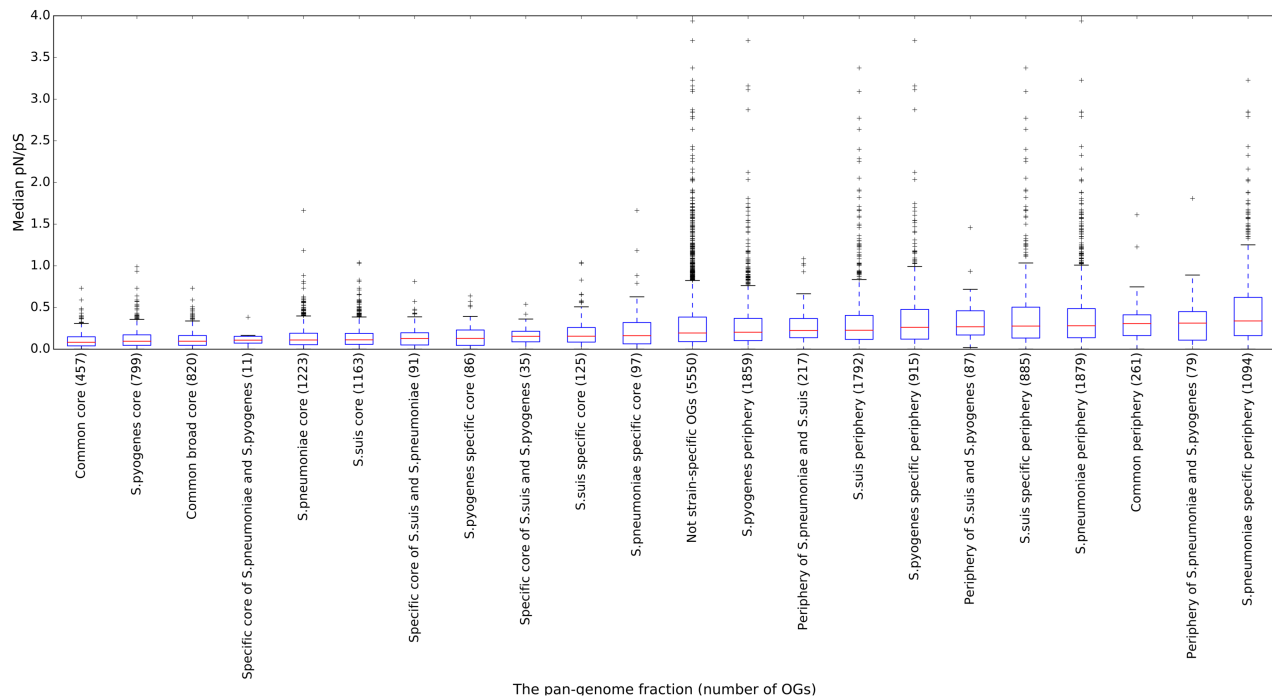

B

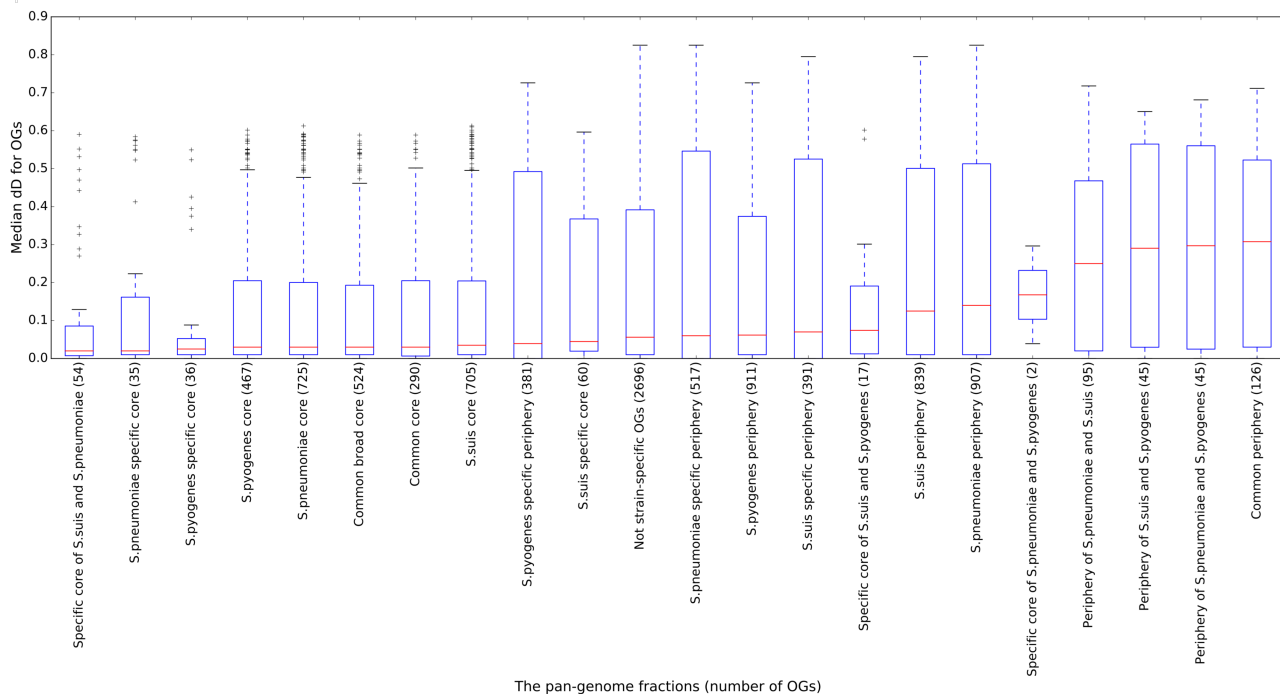

Supplement: Supplementary file 10 — Supplementary file Figure S8. Distributions of (A) the median value of the pN/pS ratio with the Jukes-Cantor correction for genes and (B) median number of nucleotide substitutions in upstream regions (dD) of genes from OGs from different pan-genome fractions. The number of analyzed OGs from each pan-genome fraction is shown in brackets. The pan-genome fractions are defined as in Fig. 4. (PDF 1248 kb) [file 12862_2019_1403_MOESM10_ESM.pdf]

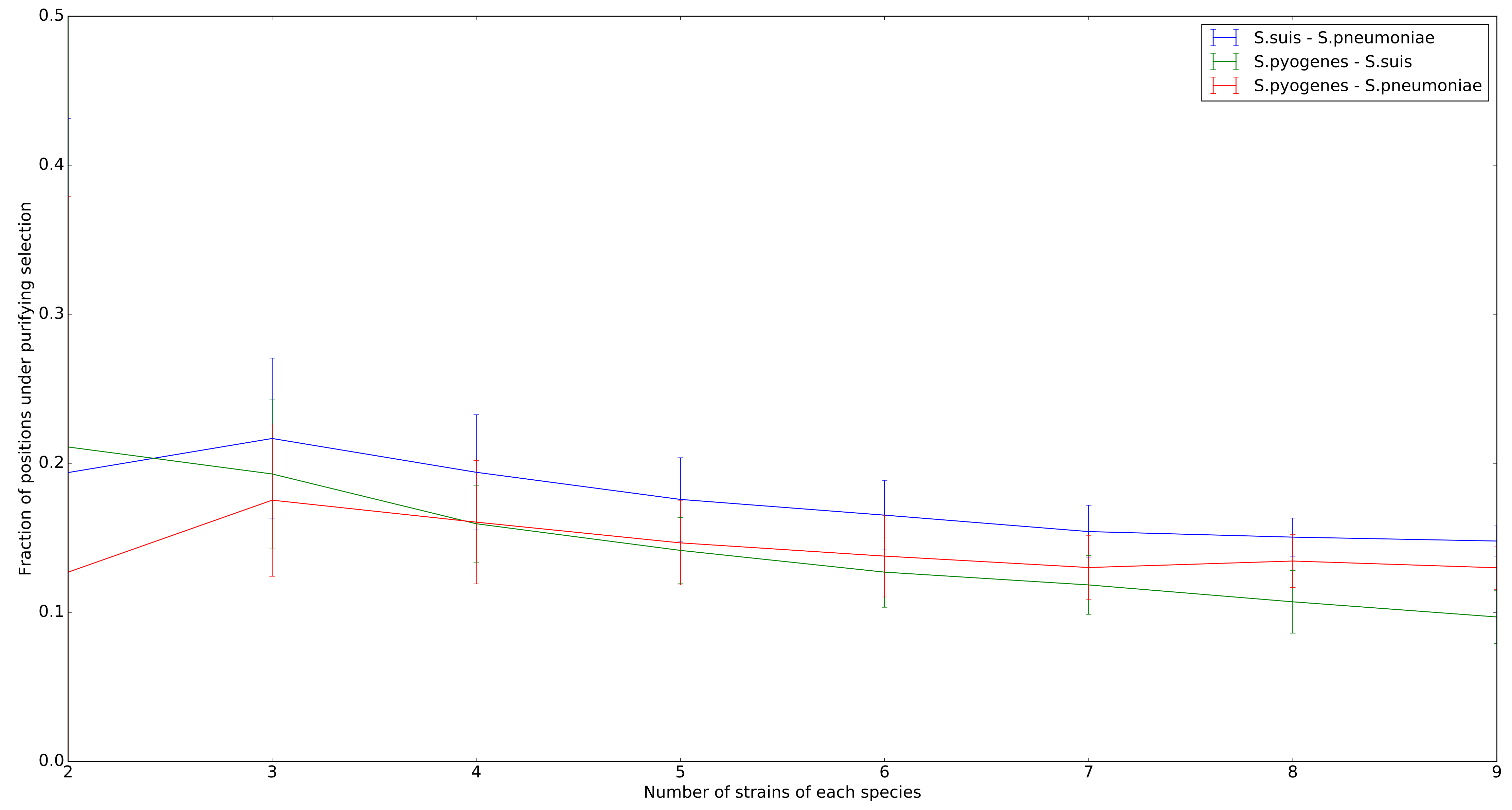

Supplement: Supplementary file 11 — Supplementary file Figure S9. Fractions of nucleotides under purifying selection in upstream fragments of core OGs as a function of the number of compared strains in pair-wise analysis of species. (PDF 17 kb) [file 12862_2019_1403_MOESM11_ESM.pdf]

**A**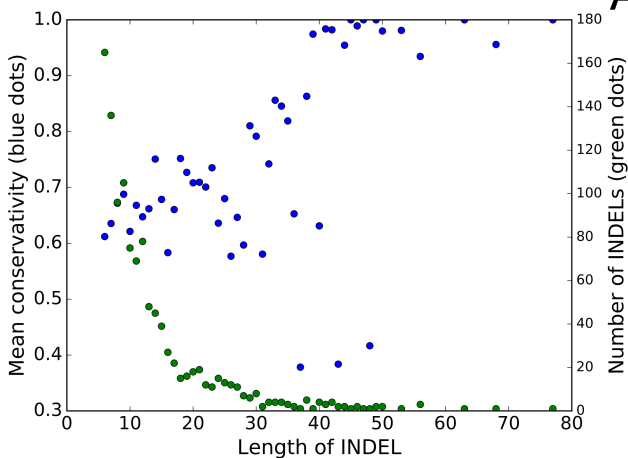**B**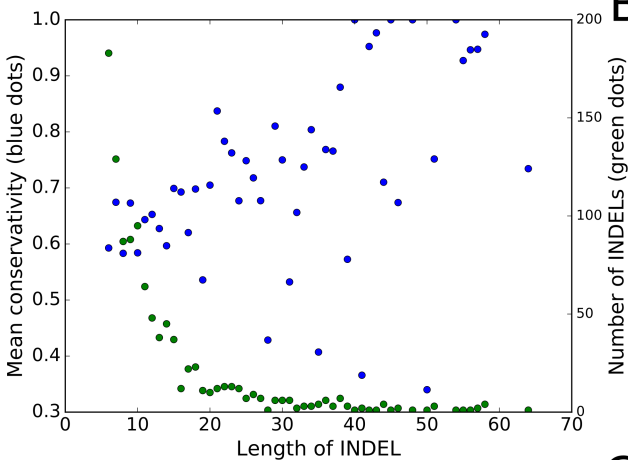**C**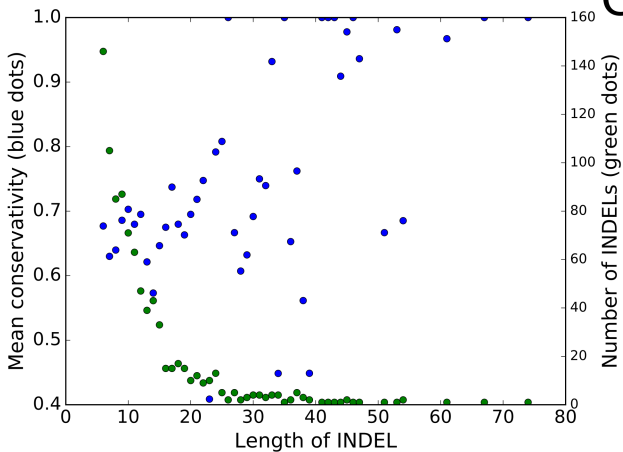

Supplement: Supplementary file 12 — Supplementary file Figure S10. Dependence of the mean conservation level of nucleotides in indels on the indel size. Blue dots correspond to the mean conservation level, green dots correspond to the number of indels of this size. (A) S. pneumoniae, (B) S. suis, and (C) S. pyogenes. (PDF 504 kb) [file 12862_2019_1403_MOESM12_ESM.pdf]

**A**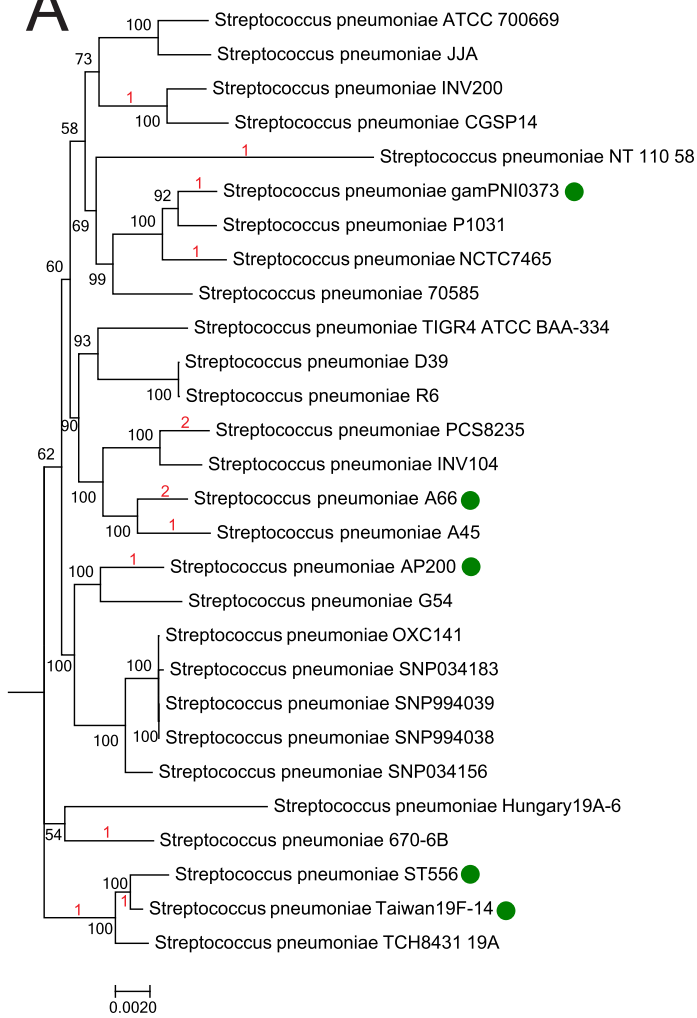**B**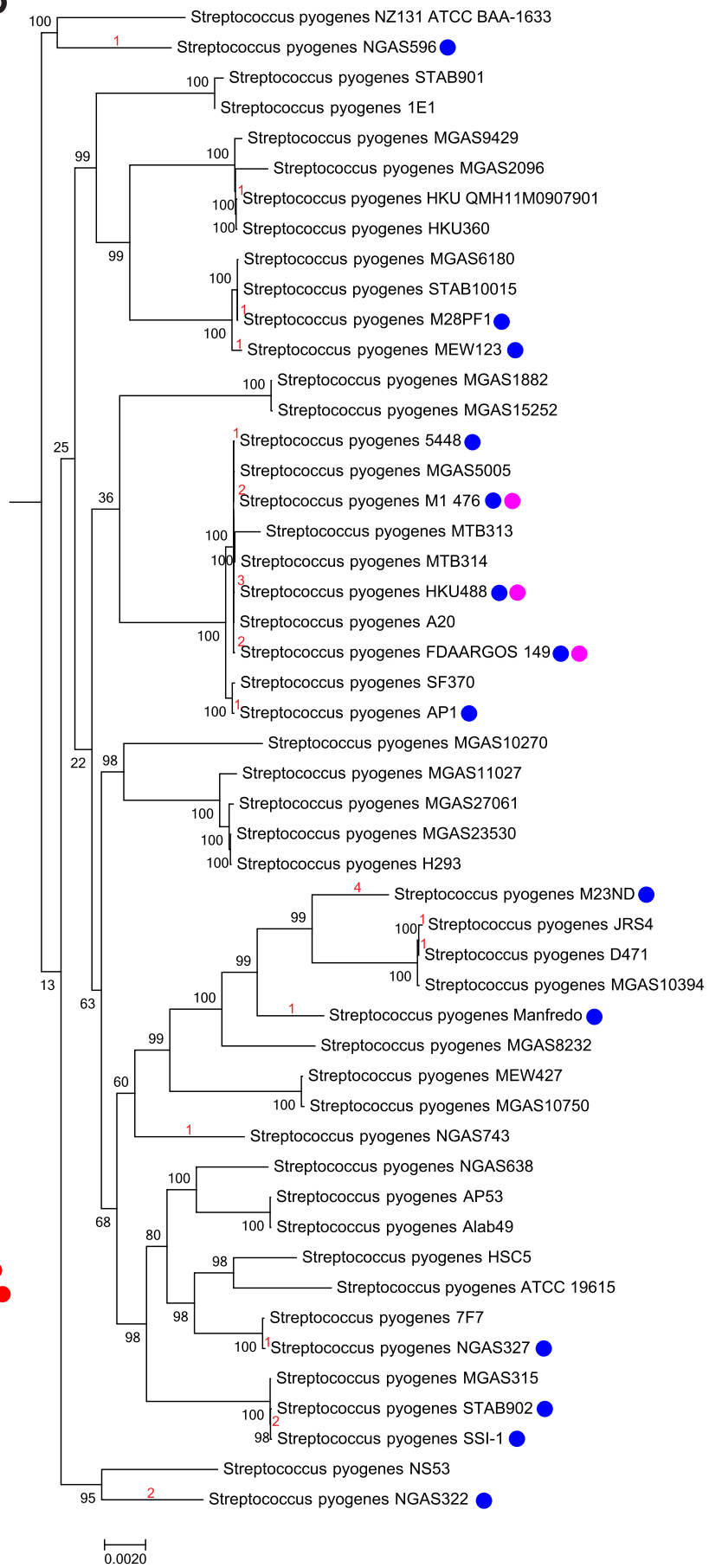**C**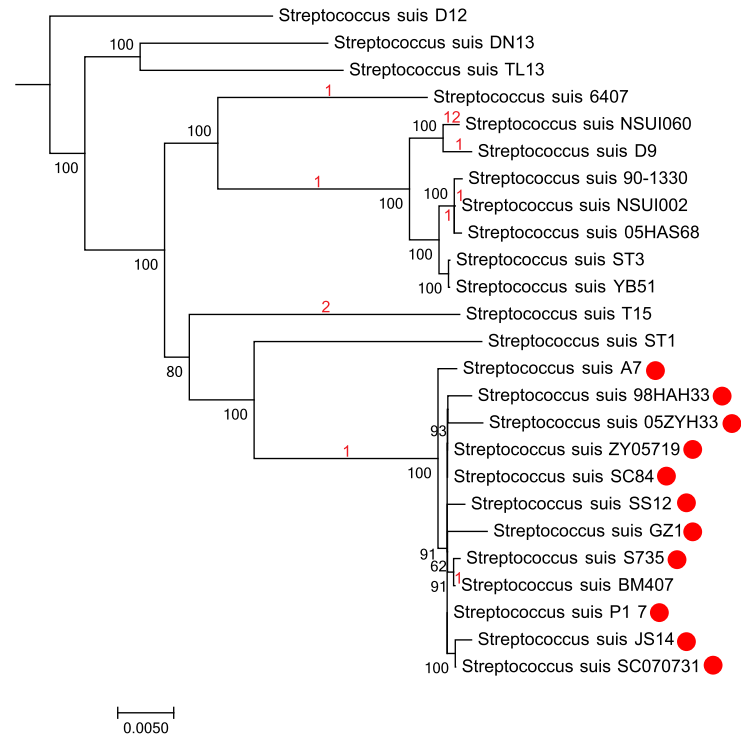

Supplement: Supplementary file 13 — Supplementary file Figure S11. Phylogenetic trees based on genes involved in parallel inversions. (PDF 867 kb) [file 12862_2019_1403_MOESM13_ESM.pdf]

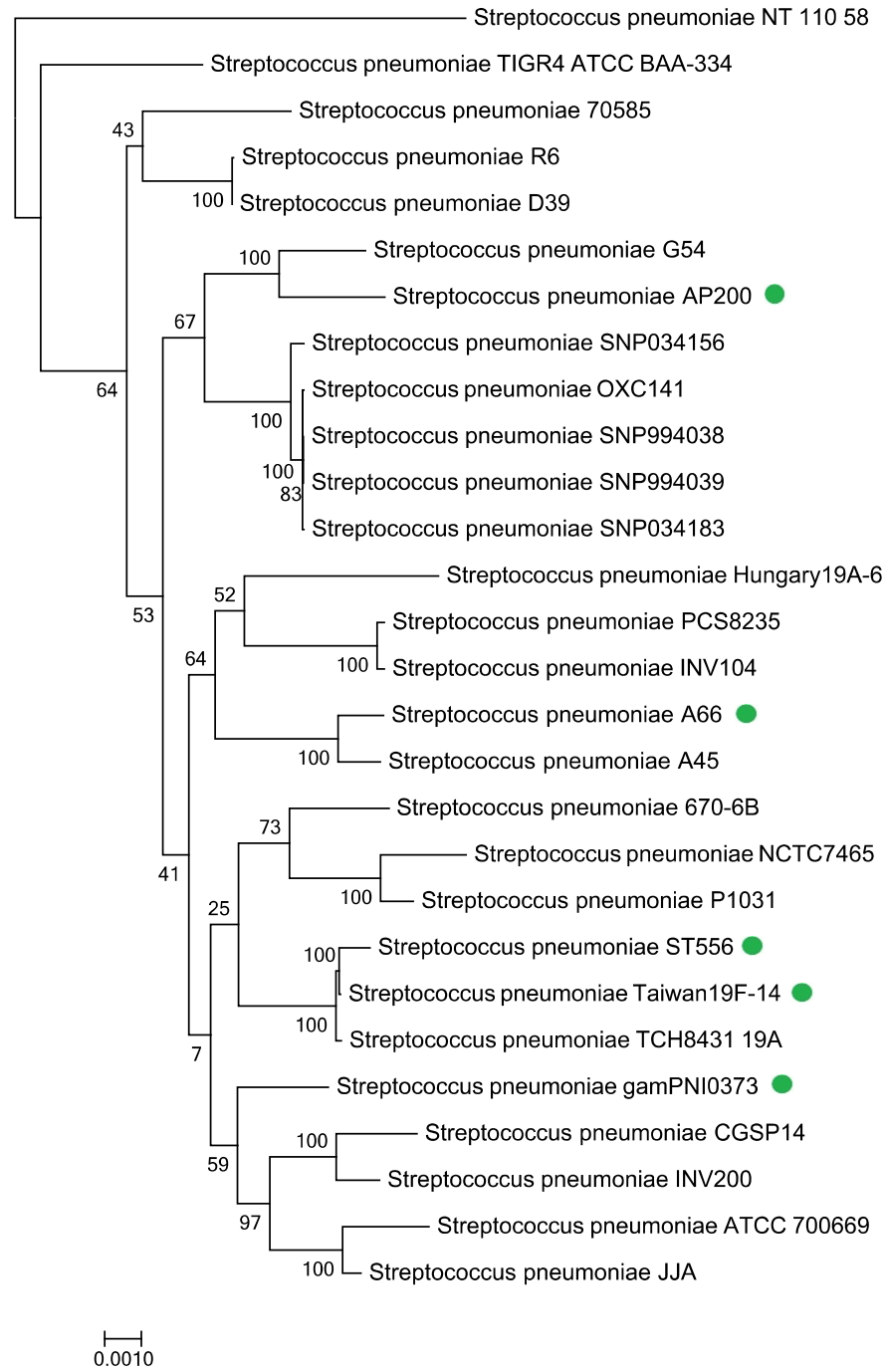

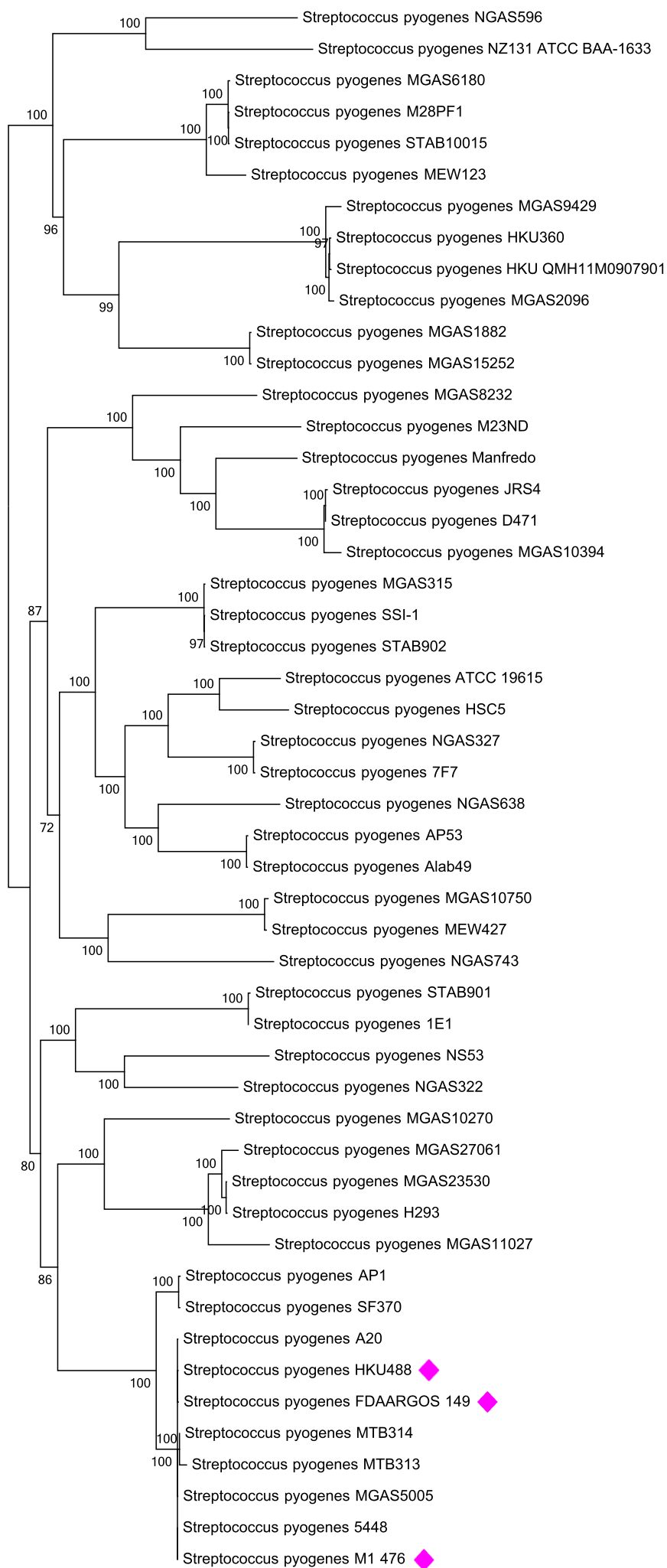

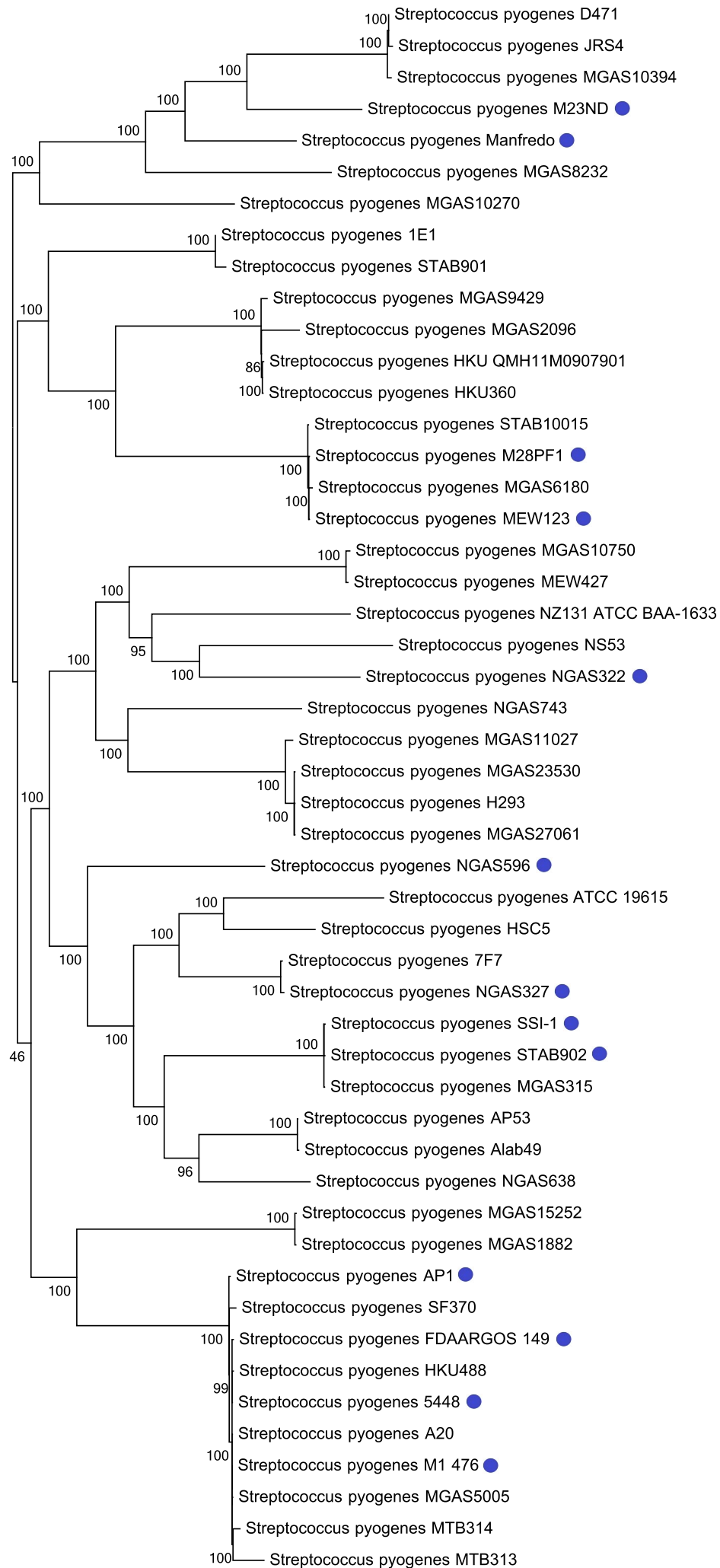

0.0010

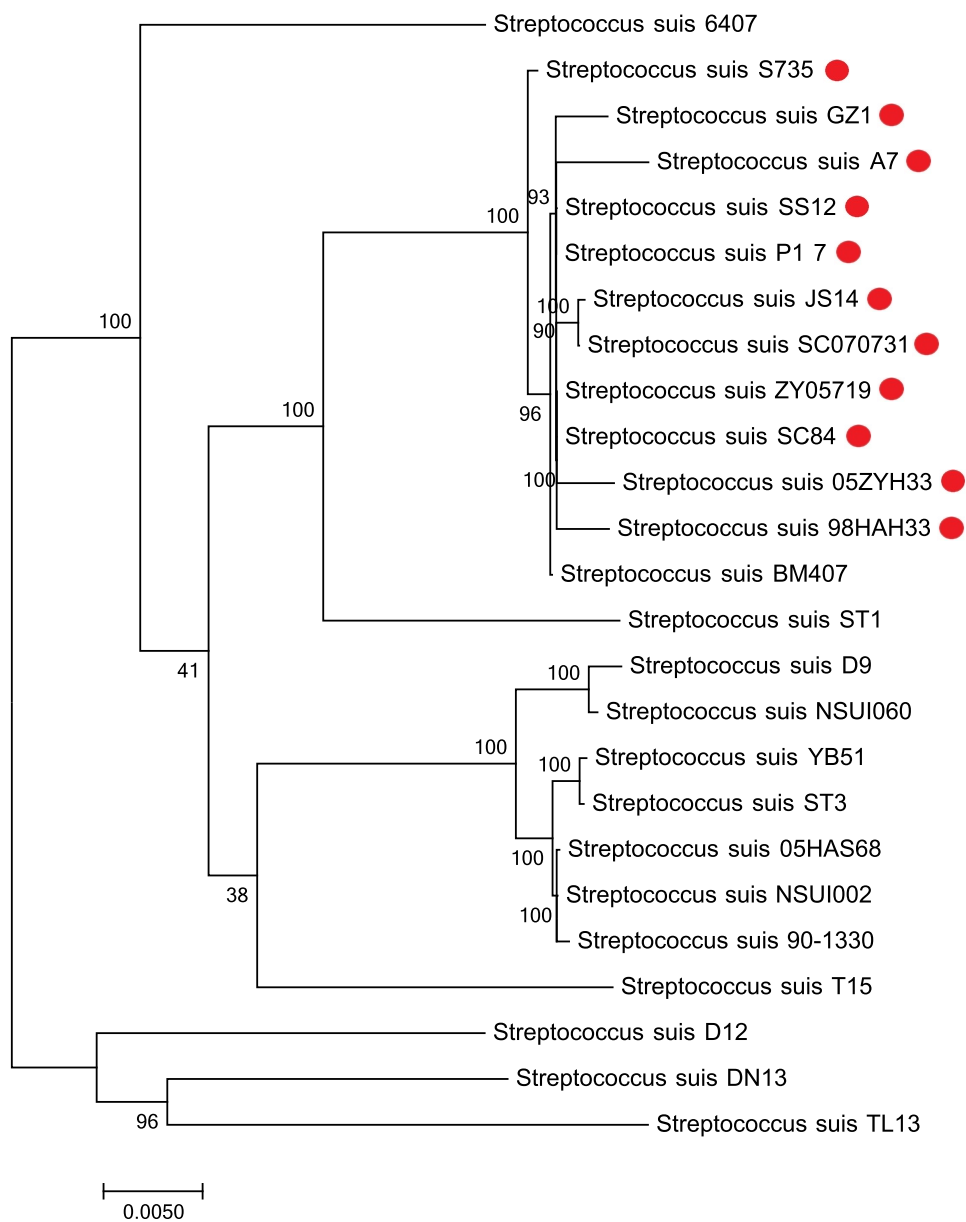

Supplement: Supplementary file 14 — Supplementary file Figure S12. Phylogenetic trees for S. pneumoniae (A), S. suis (B), S. pyogenes (C) based on the alignments of universal single-copy genes. The numbers at tree branches show the numbers of inversions. Strains with parallel inversions are marked by color labels. Strains with the same inversion are marked by the same color. (PDF 1279 kb) [file 12862_2019_1403_MOESM14_ESM.pdf]
